# Quaternary landscape evolution of Apennines peri-Adriatic belt: Insights into climate and tectonics from the fluvial record

**DOI:** 10.1126/sciadv.aec5376

**Published:** 2026-04-24

**Authors:** Valeria Ruscitto, Michele Delchiaro, Maïlys Richard, Giulia Iacobucci, Daniela Piacentini, Francesco Troiani, Marta Della Seta

**Affiliations:** ^1^ Department of Earth Sciences, Sapienza University of Rome, Rome, Italy.; ^2^ Centre national de la recherche scientifique Archéosciences Bordeaux, Bordeaux Montaigne University, Bordeaux, France.; ^3^ Department of Early Prehistory and Quaternary Ecology, University of Tübingen, Tübingen, Germany.

## Abstract

Reconstructing the landscape evolution of the Apennines peri-Adriatic belt requires separating climatic and tectonic signals within its fluvial terrace record, a task so far limited by sparse chronological constraints. Here, we mapped terrace features from three rivers draining the peri-Adriatic belt by combining semiautomatic extraction of tread surfaces from high-resolution digital terrain models with detailed mapping of associated basal strath surfaces using the 1:10,000 geological map. Nine luminescence and radiocarbon ages, integrated with sedimentological data and published chronologies, refine the timing of terrace formation for the region. Although age uncertainties are substantial, cumulative probability distributions indicate that many terrace deposits broadly coincide with late-interglacial cooling and glacial conditions, whereas older terraces show more dispersed ages. Age-elevation modeling reveals spatially variable uplift histories since ~1 million years ago, consistent with differential crustal uplift or long-strike variations in anticline growth. Overall, these results provide new constraints on the interplay between climate and tectonics shaping the peri-Adriatic landscape.

## Teaser

Quaternary landscape evolution of the Apennines peri-Adriatic belt revealed by fluvial terraces and climate-tectonic interplay.

## INTRODUCTION

The long- and short-term evolution of fluvial systems reflects a dynamic interplay between external forcings, such as climate and tectonics, and internal channel processes (*1*, *2*). On short timescales, fluvial morphology evolves around bankfull conditions, the discharge at which rivers most efficiently transport sediment and reshape their floodplains (*3*, *4*). This evolution is closely tied to modern channel dynamics and floodplain activity (*5*). In contrast, long-term evolution, as archived in fluvial terraces, offers a geological record of incision and aggradation episodes that span tens to hundreds of thousands of years. While the short-term bankfull regime filters climatic variations, fluvial terraces provide a cumulative record of how those signals and tectonic processes have shaped the landscape over time (*6*).

Fluvial terraces are relict landforms representing abandoned floodplains and constitute key archives for reconstructing past landscape dynamics (*7*). Each terrace is bounded by a tread surface at the top and a strath surface at the base, and they are commonly classified into two end-member categories [sensu (*8*)]: (i) strath terraces, which consist of planar erosional surfaces with a thin sedimentary cover; and (ii) fill terraces, which are characterized by a substantially thicker alluvial sequence. Because strath terraces are erosional surfaces with a limited sedimentary record, they are typically used in tectonic geomorphology to quantify incision and surface uplift rates. Conversely, the more extensive deposits of fill terraces are better suited to reconstruct climatic fluctuations and sedimentary dynamics.

However, the challenge in using fluvial terraces lies in the complexity of the signals preserved within them. Terrace formation results from fluctuations in the sediment supply-to-water discharge ratio (*Q*_S_*/Q*_W_) (*9*), which is, in turn, modulated by climate and tectonics (*10*).

Numerous examples are present in literature demonstrating the control of climate on fluvial terraces formation [(*11*) and references therein]. Climatic changes influence *Q*_W_ via precipitation variability and ice melting (*12*) and *Q*_S_ by affecting regolith production (*13*) and vegetation cover (*14*) and by modifying the sediment supply from hillslopes (*15*).

Sequences of fluvial terraces also reflect variations in river base level. These shifts are driven not only by climate-induced eustatic sea level fluctuations (*16*) but also by tectonic processes, especially surface uplift and fault activity (*17*). In this context, tectonics contribute to *Q*_*S*_ by accelerating erosion through the steepening of slopes and the increase of hillslope instability (*18*, *19*). Moreover, uplift raises catchment relief and stream power, enhancing the capacity of rivers to incise and mobilize sediment while also periodically resetting base levels and reorganizing drainage networks [e.g., (*20*, *21*)].

Interpreting the stratigraphic archive of terraces additionally requires consideration of stratigraphic incompleteness. Over longer integration timescales, the likelihood of stratigraphic hiatuses increases (*22*), and the derived rates become increasingly sensitive to the measurement interval (*23*). Hiatuses commonly result from rare, high-magnitude events governed by heavy-tailed distributions of discharge and sediment flux. These stochastic events do not follow exponential decay but exhibit non-negligible tail probabilities, meaning that infrequent but powerful floods can dominate sedimentary signals (*24*). In general, river incision is typically promoted by an increase in discharge relative to sediment supply, whereas sediment accumulation occurs when supply outpaces transport capacity, leading to deposition in the floodplain and valley widening (*25*). Additional complexities arise from variations in bedrock lithology (*26*) and human activities such as gravel extraction, river channelization, and damming (*27*). Moreover, not all landscape changes result from external forcings: Autogenic feedbacks within fluvial systems can generate intrinsic, nonlinear dynamics that obscure or overwrite external climatic and tectonic signals (*28*). Disentangling these overlapping controls remains a central challenge in terrace-based reconstructions (*29*).

**Fig. 1.**
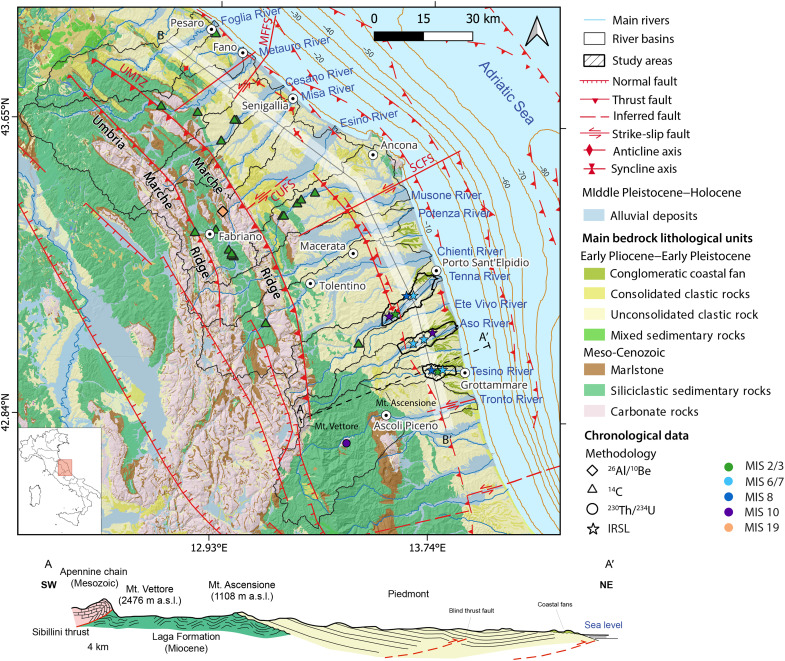
Overview of the study area together with chronology available from literature and this study. Lithological map of the central Apennines of Italy (*108*), with the location of the ages relative to fluvial deposits available in literature and reported in detail in table S1. Star symbol indicates infrared stimulated luminescence (IRSL) ages. The reported faults are taken from the Individual Seismogenic Sources from the Database of Individual Seismogenic Sources (DISS) catalog [version 3.3.0 (*109*)] and from (*44*): SCFS (South Conero Fault System), MFFS (Metauro-Fano Fault System), and CUFS (Cupramontana Fault System). The hillshade is derived from the 1–arc sec Shuttle Radar Topography Mission Digital elevation Model (DEM) (*110*). The coordinate system is WGS84.

In this context, we investigated the well-preserved staircase of fluvial fill terraces in the peri-Adriatic belt of the Apennines (Italy) to reconstruct landscape evolution from Middle Pleistocene and to provide new insights into the roles of climate and tectonics in controlling fluvial sedimentation. We produced new chronological constraints using infrared stimulated luminescence (IRSL) and radiocarbon dating for nine terrace deposits across the Tesino, Aso, and Tenna river basins, and we conducted an extensive review of all previously published terrace ages in the region, including related chronologies from speleothems and travertines (Fig. 1 and table S1).

Since 1968, four major levels of fill terraces have been recognized, described, and dated along the peri-Adriatic belt. The fourth level corresponds to the modern floodplain and is not considered in this study. Beginning in the north, Mencucci *et al.* (*30*) reported a radiocarbon age of 23.5 ± 0.12 thousand years (ka) for the third-level terrace of Foglia River. For the Metauro River, Calderoni *et al.* (*31*) obtained two radiocarbon ages older than 43 to 44 ka for the third-level deposits. In the Cesano river basin, Calderoni *et al.* (*32*) reported five radiocarbon ages for the third level ranging from 31.7 ± 1.05 to 37.3 ± 2.2 ka. For Esino River, radiocarbon ages between 14.7 ± 0.15 and 41 ± 4 ka were obtained in (*33*, *34*) and (*35*) for the third-level terrace. Additional evidence from the Esino basin includes a ^26^Al/^10^Be burial age of 750 ± 260 ka for fluvial sediments in the Grotta della Madonna cave at Frasassi Gorge (*36*). For Musone River, Wegmann and Pazzaglia (*37*) constrained the third-level terrace to between 16.73 ± 0.09 and 30.98 ± 0.24 ka. Along the Chienti River, (*38*) obtained radiocarbon ages of 26.8 ± 0.7 and 30.15 ± 1.2 ka for the same terrace level. In the Tenna River basin, Damiani and Moretti (*39*) reported two radiocarbon ages of 20.02 ± 0.15 and >44 ka for third-level deposits. Last, Sembroni *et al.* (*40*) applied ^230^Th/^234^U dating to eleven travertine samples from three stratigraphic levels; only one sample, associated with a first-level fluvial conglomerate, yielded an age >350 ka.

By generating new chronological constraints on the first, second, and third terrace levels across the studied basins (Fig. 1), we dated back to the Middle Pleistocene the landscape evolution of the peri-Adriatic belt area, offering new insights on the timing and drivers of sediment deposition and thereby inferring the roles of climate and tectonics in shaping the fluvial architecture of the region.

### Geological setting

The study area constitutes the foreland of the arcuate northeast-verging fold-and-thrust orogenic belt of the Central and Northern Apennines. This orogenic belt is the result of the convergence between several blocks belonging to Eurasia and African plates, which starting from the Cretaceous formed first the Alpine orogenic system, followed then in the Neogene by the Apennines chain (*41*). During the Late Oligocene to Early Miocene, the Apennines accretionary prism was activated, and thrust sheets propagated eastward toward the foreland (*42*). Evidence of this dynamics is represented by two ridges parallel to the coastline: the Umbria-Marche Ridge and the Marche Ridge, respectively, from west to east. The aforementioned ridges are separated to the east from the foreland by the Umbria-Marche thrust zone (UMTZ; Fig. 1). The thrust sheets mainly involve the Mesozoic-Paleogene Umbria-Marche sedimentary succession characterized by a preorogenic Triassic sequence of carbonates, followed by deeper sea succession.

Moreover, a series of northeast-southwest transversal structures, such as the South Conero Fault System (SCFS) and the Metauro-Fano Fault System (MFFS), characterize the peri-Adriatic sector (Fig. 1). Some authors (*43*, *44*) suggested that these structures were inherited from an earlier extensional phase and then reactivated during the compressive phase as left lateral strike-slip faults.

During the Late Pliocene to Early Pleistocene, the backarc extension associated to the rollback of the subduction slab (*45*) brought to the cross-cut of the previously formed orogenic prism and to the development of a contracting east side and an extensional west side moving coupled eastward (*46*, *47*). The extension is mainly expressed along southwest-dipping normal faults, which originate local range uplift and opening of intramontane basins [e.g., (*48*, *49*)] The onset of normal faulting follows a north-eastward younging trend in the Northern Apennines (*50*). In the Central Apennines, in contrast, normal faults initiated almost synchronously at about 2.5 million years ago (Ma) (*51*).

In the Apennines peri-Adriatic belt, the seismic activity is mostly attributed to the orogen-parallel thrust faulting, such as the following: the equivalent magnitude 5.3 Senigallia earthquake of 1930 (*52*), the equivalent magnitude 5.8 Offida earthquake of 1943 (*53*), the equivalent magnitude 4.6 Ancona earthquake in 1972 (*54*), the moment magnitude (*M*_w_) 5.1 Porto S. Giorgio earthquake of 1987 (*55*), and the *M*_w_ 5.5 Fano-Pesaro earthquake of 2022 (*56*). In this regard, several studies suggested that blind thrust faults, accompanied by anticlinal growth, characterize the active tectonics of the Marche coastal area and the adjacent Adriatic offshore [e.g., (*57*)]. According to this view, blind thrust faults would be responsible for the occurrence of the largest earthquakes in the Adriatic coastal area. However, strike-slip seismicity in the anti-Apennine (northeast-southwest) direction is also recorded (*58*, *59*).

The peri-Adriatic belt is characterized by a turbiditic terrigenous sequence formed between the Messinian and Early Pliocene (*60*). Since the Middle to Late Pliocene, the area experienced a regional uplift responsible for the gradual emersion of the area, during which the sedimentation evolved from marine to shallow-water deltaic settings (*61*). This sequence consists of two distinct formations: the Argille Azzurre Formation (Fm.) (Pliocene to Lower Pleistocene) and the Fermo Fm. (Lower Pleistocene). These formations are characterized by varying lithologies transitioning from pelites and sands at the base to intercalated layers of arenaceous and conglomeratic bodies forming the top of the sequence, which are associated with submarine fans, also known as “fanglomerates” according to Nesci and Savelli (*62*).

From Middle Pleistocene, the prolonged uplift with rates ranging from 0.3 to 0.5 mm year^−1^ (*63*, *64*) led to the development of equally spaced transverse rivers east-northeast-west-southwest oriented and flowing toward east-northeast, which transversally cut the main morphostructures (*65*) and generated staircases of fluvial terraces. In particular, four major levels of terraces of the alluvial type [fill terraces; sensu (*8*)] were recognized, hanging at different heights from a few meters up to 150 m above the present thalweg. Descriptions of these terraced deposits are present in literature (*30*–*35*, *37*–*40*, *66*–*68*), and geochronological data are available for the youngest deposits (see table S1).

## RESULTS

### Planoaltimetric distribution of the fluvial terrace staircases

The study started with a first phase dedicated to the surveying and mapping of the fluvial terraces’ features, including the upper tread and the associated alluvial deposits. The fluvial terrace tread upper surface was identified using a semiautomatic mapping procedure applied to high-resolution digital terrain models (DTMs). The associated alluvial deposits were mapped and sedimentologically described on the basis of the Quaternary deposits of the geological map 1:10,000 (available at www.regione.marche.it/Regione-Utile/Paesaggio-Territorio-Urbanistica/Cartografia/Repertorio/Cartageologicaregionale10000) and field surveys. The contact between alluvium and bedrock was interpreted as the exposure of the strath basal surface. To estimate the terrace level thickness, we analyzed the elevation above the thalweg distribution of both tread and strath surfaces.

The representative elevations of the tread and strath surfaces were taken as the 75th and 25th percentiles of their respective distributions, with the associated uncertainty defined as the difference between these values and the 50th percentile. Furthermore, we also considered the basal contact of the Fermo Fm. because it provides the upper limit on age of the river valleys. Specifically, we extracted the 50th percentile of its elevation above thalweg distribution and assumed a total thickness of 40 m (*69*). To minimize biases caused by downstream convergence of all terrace levels, including the Fermo Fm., toward the river mouth, we restricted the analysis to relative elevation values located within 6 km upstream of the outlet.

**Fig. 2.**
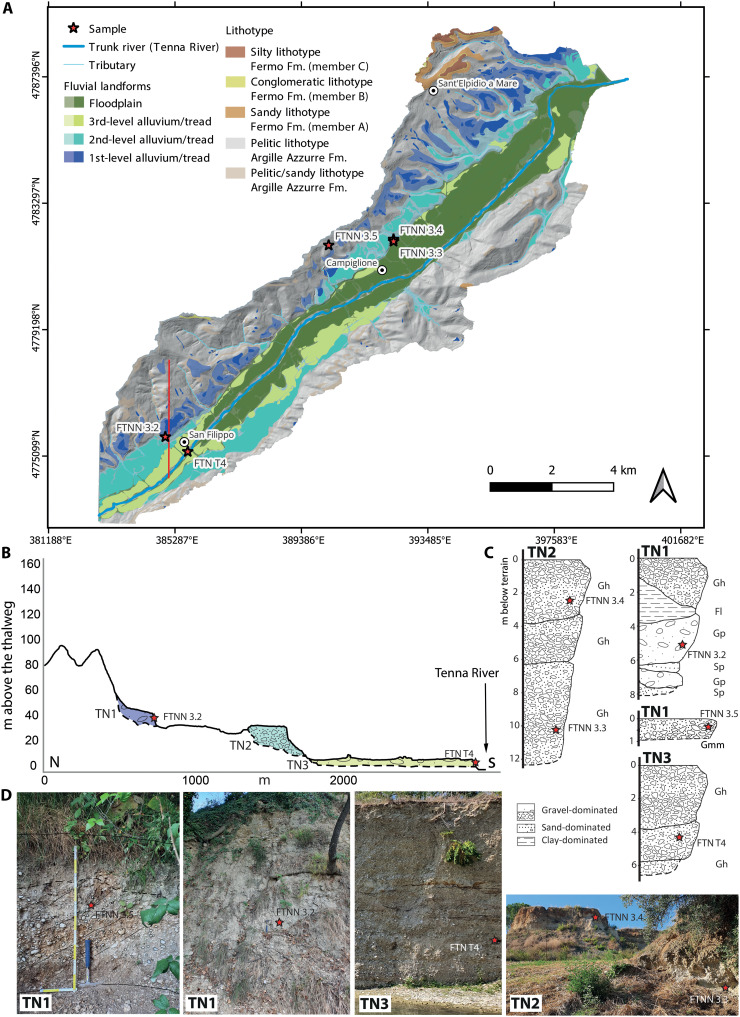
Fluvial terraces map of the Tenna River with sampling locations detailed. Map of the terraces of the Tenna River in (**A**) together with north (N)–south (S) transversal profile (**B**), simplified logs with facies [Gh, clast-supported, crudely bedded gravel; Gp, stratified gravel; Gmm, matrix-supported, massive gravel; Fl, sand, silt, mud; Sp, sand, fine to very coarse, may be pebbly; sensu Miall (*70*)] (**C**), and photos of the sampled outcrops (**D**) (photo credit: V.R., Sapienza University of Rome).

**Fig. 3.**
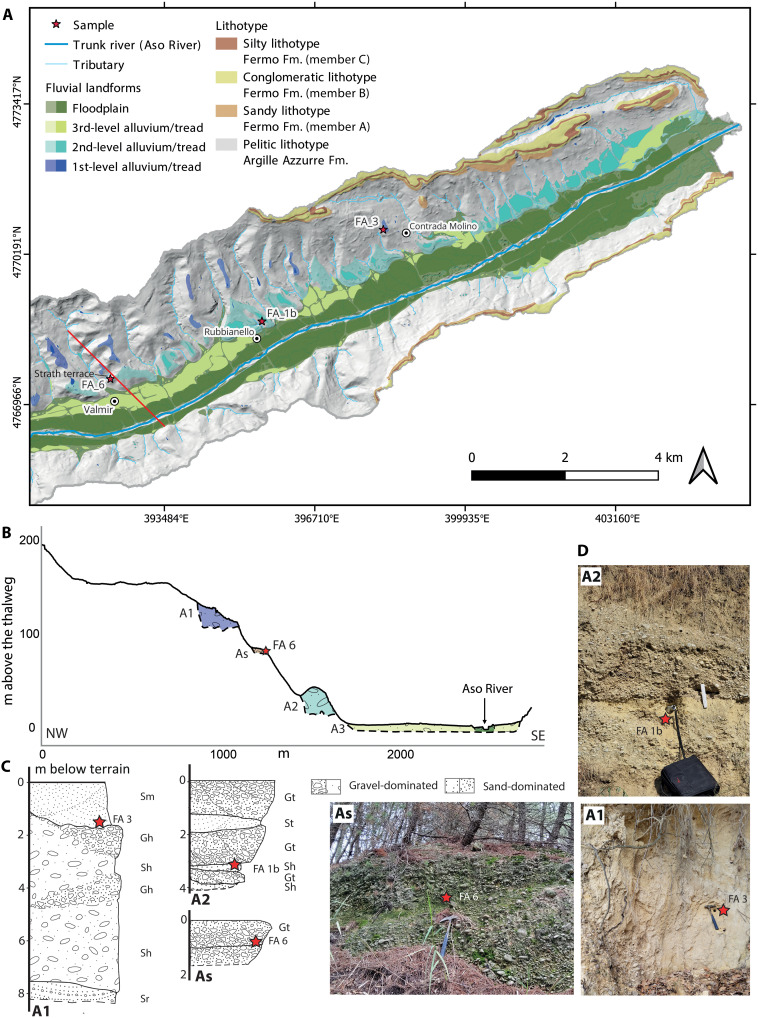
Fluvial terraces map of the Aso River with sampling locations detailed. Map of the terraces of the Aso River in (**A**) together with northwest (NW)–southeast (SE) transversal profile (**B**), simplified logs and facies [Gh, clast-supported, crudely bedded gravel; Gt, stratified gravel; Sm, fine to coarse sand; Sh, sand, very fine to coarse may be pebbly; Sr, sand, very fine to coarse; Sh, sand, fine to very coarse, may be pebbly; sensu Miall (*70*)] (**C**), and photos of the sampled outcrops (**D**) (photo credit: V.R., Sapienza University of Rome).

**Fig. 4.**
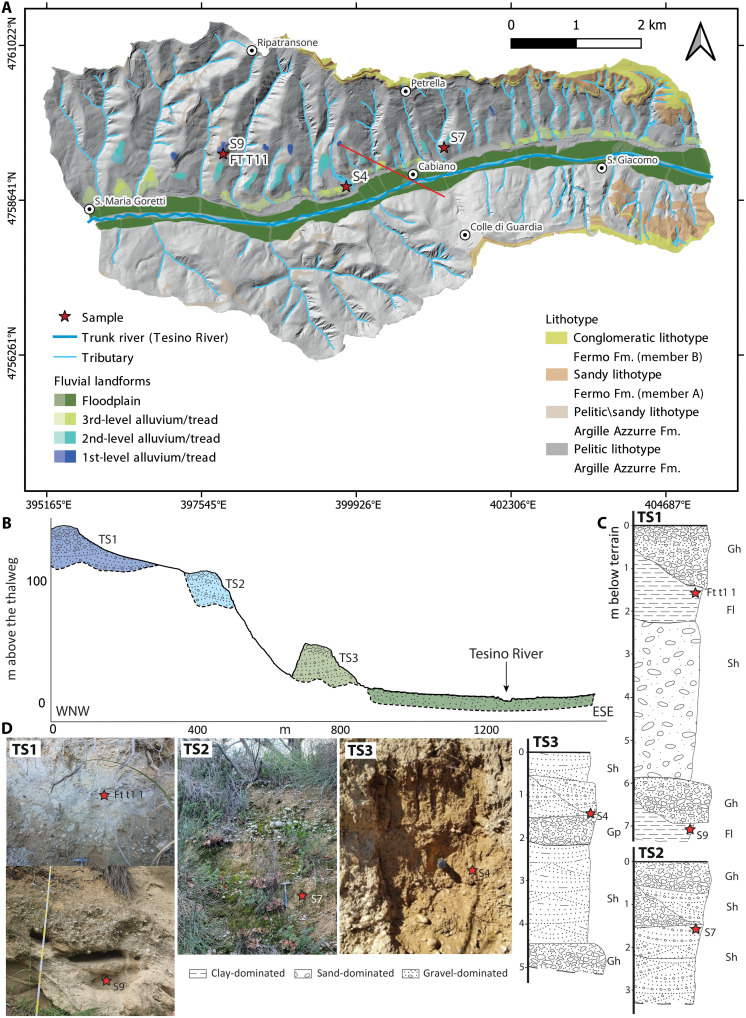
Fluvial terraces map of the Tesino River with sampling locations detailed. Map of the terraces of the Tesino River in (**A**) together with west-northwest (WNW)–east-southeast (ESE) transversal profile (**B**), simplified logs with facies [Gh, clast-supported, crudely bedded gravel; Gp, stratified gravel; Fl, sand, silt, mud; Sh, sand, very fine to coarse, may be pebbly; sensu Miall (*70*)] (**C**), and photos of the sampled outcrops (**D**) (photo credit: V.R., M.D., and GI., Sapienza University of Rome).

Nearly all of the identified terraces are fill terraces (see Figs. 2 to 4), distinguished by a thick alluvium overlying the basal strath surface. The only exception is a single strath (erosional) terrace along the Aso River, which is characterized by a relatively thin sediment cover above its basal strath surface.

#### Tenna River

**Fig. 5.**
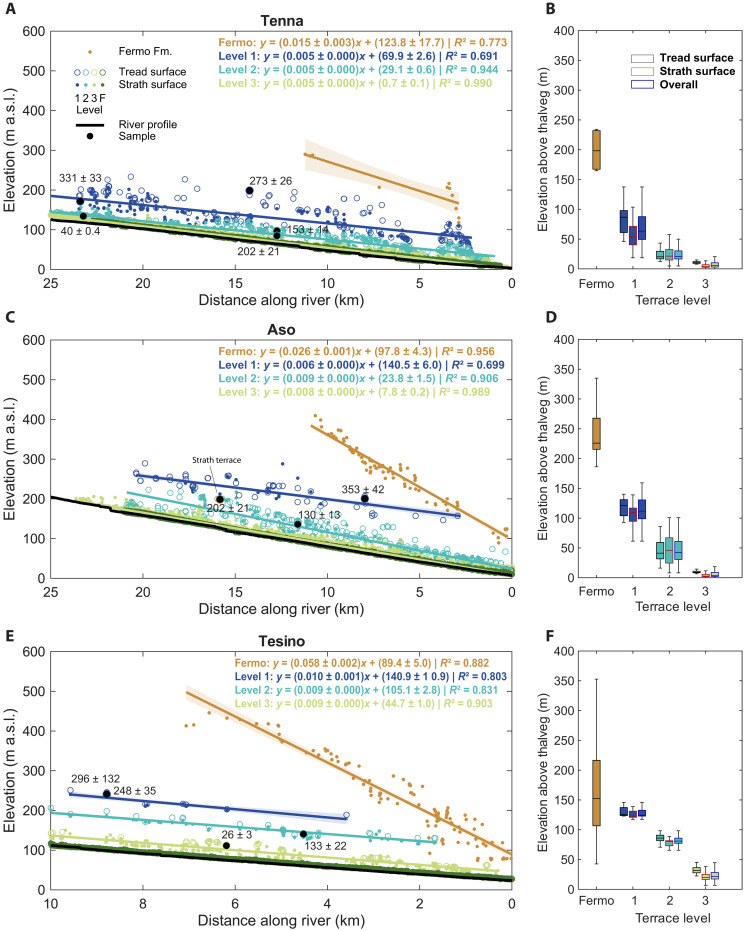
Terraces tread and strath surfaces and sample locations projected along longitudinal profiles of the three rivers. Longitudinal profiles of the (**A**) Tenna, (**C**) Aso, and (**E**) Tesino rivers, showing the elevation of terrace treads, strath surfaces, sample sites, and the base of the Fermo Fm. For each terrace level and for the Fermo Fm., regression lines and their confidence intervals are displayed, along with linear fit parameters and coefficients of determination (*R*^2^). Box plots illustrating the distribution of elevation above the thalweg for treads, straths, and the combined dataset are shown for the (**B**) Tenna, (**D**) Aso, and (**F**) Tesino rivers. F, floodplain.

For the Tenna River, three terrace levels have been identified, mainly localized on the left-hand side of the river basin (Fig. 2). As also reported in Fig. 5, the highest level, hereafter named as first, lies between 49.3 ± 14 and 87.7 ± 24.4 m above the thalweg. The intermediate terrace level, hereafter called second, ranges between 16.6 ± 4 and 30.2 ± 9.6 m above the thalweg. The lowest level, hereafter named third, reaches at its highest 10.2 ± 4.3 m above the thalweg and develops mainly into the active floodplain with its bottom at 1 ± 4.9 m. Consequently, its deposits are interbedded with the active floodplain (Fig. 2A).

From the longitudinal river profile (Fig. 5A) upon which the terrace tread and strath surfaces are projected, it can be observed that the first level is characterized by a slope of 0.005 ± 0.000, and a *y*-axis intercept value is equal to 69.9 ± 2.6 m above sea level (a.s.l.). The second-level slope is 0.005 ± 0.000, and the *y*-axis intercept is 29.1 ± 0.6 m a.s.l. Last, the third level shows an identical longitudinal slope of 0.005 ± 0.000, while the *y*-axis intercept is of 0.7 ± 0.1 m a.s.l. As for the Fermo Fm., the median elevation value above thalweg was recorded at 198.2 ± 20 m, the slope is 0.015 ± 0.003, and the intercept is 123.8 ± 17.7.

As reported in the stratigraphic logs (Fig. 2C) and the photos (Fig. 2D), the first-level terrace deposit of the Tenna River (TN1) is complex and composed of several distinct units, with alternating fine-grained and coarse-grained sections coarsening upward. According to the classification by Miall (*70*), the first terrace deposit shows an alteration of different facies from finer [facies Fl (sand, silt, mud)] to coarser ones [facies Gmm (matrix supported, massive gravel); see Fig. 2C]. The second (TN2) and third (TN3) levels instead are mainly coarse grained and well developed, and in some cases, the clasts exhibit imbricated sedimentary structures. The third and second levels can be associated with Gh facies [clast-supported, crudely bedded gravel; sensu (*70*)].

#### Aso River

In the Aso River, the highest terraces, hereafter named as first, occur from 98.9 ± 12.8 to 130.6 ± 18.9 m above the thalweg. The second level lays between 30.7 ± 11.9 and 61 ± 18.4 m above the thalweg. The third level is located between 2.1 ± 1.3 and 8.7 ± 5.3 m above the thalweg (Figs. 3 and 5).

The first-level terrace tread and strath surfaces are distributed with a slope of 0.006 ± 0.000. The second and third levels have similar slope values of 0.009 ± 0.000 and 0.008 ± 0.000 (Fig. 5B). The *y*-axis intercepts of the terrace levels are located at 140.5 ± 6 m a.s.l. for the first level, 23.8 ± 1.5 m a.s.l. for the second level, and lastly 7.8 ± 0.2 m a.s.l. for the third level. As reported in the stratigraphic logs (Fig. 3C) and the photos (Fig. 3D), the first-level terrace deposit of the Aso River (A1) is well developed, with a 1.5-m-thick fine-grained upper portion, followed by coarser deposits developing for 6 to 7 m. According to the classification by Miall (*70*), the A1 deposit shows a characteristic fine-grained deposit on top [facies Sm (fine to coarse sand)] laying over coarser deposits [facies Gh (clast-supported, crudely bedded gravel)]. The second-level terrace deposit (A2) is characterized by coarse-grained sections intercalated by fine-grained lenses. Here, a succession of asymmetrical channel belt bodies, characterized by epsilon cross-bedding, constitutes the facies Gt [stratified gravel; sensu (*70*)] (see Fig. 3C). For the third-level terrace, a consistent deposit well suited for sampling and sedimentological characterization was unfortunately not identified. As for the Fermo Fm., the median elevation value above thalweg was recorded at 225.8 ± 20 m, the slope is 0.026 ± 0.001, and the *y*-axis intercept is 97.8 ± 4.3 m a.s.l.

During the phase of identification of the deposits on the field, another type of terrace has been identified (As), laying from 73 to 76 m above the thalweg. This was characterized by a thin deposit, with a thickness of around 3 m. The deposit is homogeneous, clast supported, coarse grained with a sandy matrix, and directly carved on Argille Azzurre Fm. On the basis of these characteristics, it was identified as a strath terrace deposit.

#### Tesino River

For the Tesino River, three terrace levels have been identified. The first level ranges from 122.9 ± 1.4 to 132.7 ± 8.4 m, the second level from 77.2 ± 3.7 to 85.9 ± 5 m, and lastly the third level from 17.3 ± 4.1 to 28.3 ± 6.8 m above the thalweg (Figs. 4 and 5). The first-level terrace shows a slope of 0.01 ± 0.001 and a *y*-axis intercept at 140.9 ± 10.9 m a.s.l. The slope of the second and third terrace levels is the same with a value of 0.009 ± 0.000. The *y*-axis intercept of the second level is at 105.1 ± 2.8 m a.s.l. The third level is at 44.7 ± 1 m a.s.l. Two main types of deposits have been identified and reported in (*67*): The first one is characterized by cross-stratified gravel, moderately sorted, and well rounded, with occasional sand-dominated layers, where a coarsening upward succession can be locally observed. The second one is clast supported and lithologically heterogeneous with subrounded and poorly sorted pebbles; well bedded-laminated and sandy-mud levels can occasionally be observed. The outcrop of the first-level terrace (TS1) reaches a considerable overall thickness, preserving the clast-supported deposit [facies Gh (clast-supported, crudely bedded gravel)] according to Miall’s classification (*70*) with thick intercalations of fine-grained sections [facies Sh (sand, very fine to coarse, may be pebbly); and facies Fl (sand, silt, mud)]. The second-level terrace (TS2) presents both the deposits, intercalated, with alternations between the facies Gh and Sh [sensu (*70*)]. Last, the third-level terrace deposit (TS3) presents cross-stratified deposits at the top [facies Gp (stratified gravel)] while at the bottom are finer deposits (facies Sh).

As for the Fermo Fm., the median elevation value above thalweg was recorded at 152 ± 20 m, the slope is 0.058 ± 0.002, and the *y*-axis intercept is 89.4 ± 5 m a.s.l.

### Grain size analysis and statistics

While the sedimentological analysis, whose results are reported in the previous sections, aimed to assess qualitatively the different depositional facies and to differentiate the strictly alluvium deposits from the colluvial ones, the grain size analysis and the statistical grain size distribution helped to reconstruct the depositional flow conditions associated with each terrace level. We used a combined methodological approach that included photoanalysis (for the coarser fraction) and laboratory sieving (for the finer fraction) to ensure a comprehensive characterization of the heterogeneous nature of grain size distribution in fluvial deposits.

Grain size analyses of sediment samples from the Aso, Tenna, and Tesino rivers, based on photoanalysis and sieving methods and their combination, reveal distinct sedimentological characteristics across the three fluvial systems. The main results are reported in fig. S1 (see the Supplementary Materials) and Table 1.

**Table 1.** Grain size statistics.

Combined							
River	Level	Sample #	Mean ϕ	Mean diameter (mm)	Standard deviation	Skewness	Kurtosis
Tenna River	1st	TN1	−1.78	3.44	3.07	0.68	0.55
	2nd	TN2	−2.71	6.54	2.32	0.64	1.10
	3rd	TN3	−3.38	10.41	1.17	0.46	2.61
Aso River	1st	A1	−2.02	4.06	3.20	0.71	0.68
	2nd	A2	−0.93	1.91	3.16	0.20	0.56
	Strath	As	−2.28	4.87	2.34	0.67	1.07
Tesino River	1st	TS1	−2.17	4.51	2.67	0.69	1.31
	2nd	TS2	−2.86	7.27	2.29	0.60	3.36
	3rd	TS3	−2.63	6.20	2.99	0.55	1.14
**Photoanalysis**							
River	Level	Sample #	Mean ϕ	Mean diameter (mm)	Standard deviation	Skewness	Kurtosis
Tenna River	1st	TN1	−3.87	14.59	0.55	−0.47	1.11
	2nd	TN2	−3.88	14.69	0.55	−0.44	1.08
	3rd	TN3	−3.74	13.39	0.40	−0.38	0.97
Aso River	1st	A1	−4.00	16.03	0.64	−0.39	0.95
	2nd	A2	−3.80	13.91	0.50	−0.50	1.07
	Strath	As	−3.72	13.16	0.40	−0.41	1.11
Tesino River	1st	TS1	−3.77	13.64	0.49	−0.45	1.21
	2nd	TS2	−3.91	14.99	0.59	−0.51	1.05
	3rd	TS3	−4.11	17.26	0.82	−0.62	0.90
**Sieving**							
**River**	**Level**	**Sample #**	**Mean ϕ**	**Mean diameter (mm)**	**Standard deviation**	**Skewness**	**Kurtosis**
Tenna River	1st	TN1	0.59	0.66	2.77	−0.64	0.57
	2nd	TN2	−0.94	1.92	2.51	0.23	0.84
	3rd	TN3	−3.32	9.97	1.85	0.39	1.15
Aso River	1st	A1	1.02	0.49	2.50	−0.36	0.69
	2nd	A2	1.10	0.47	2.37	−0.37	0.70
	Strath	As	−1.59	3.01	2.70	0.39	0.70
Tesino River	1st	TS1	−1.42	2.68	3.19	0.39	0.66
	2nd	TS2	−2.27	4.81	3.33	0.47	0.66
	3rd	TS3	−0.89	1.85	3.34	0.15	0.64

#### Tenna River

The Tenna River samples TN1, TN2, and TN3, corresponding respectively to the first, second, and third levels, display the coarsest overall texture among the three rivers. Mean diameters range from 3.44 mm (TN1) to 10.41 mm (TN3), with corresponding mean φ values from −1.78 to −3.38. Sorting improves with increasing coarseness, ranging from poorly sorted (3.07 in TN1) to moderately sorted (1.17 in TN3). Skewness is consistently positive (0.46 to 0.68), and kurtosis ranges from 0.55 to 2.61, indicating variability with TN3 being notably leptokurtic.

The coarse fraction is highly uniform across Tenna samples, with mean diameters between 13.39 and 14.69 mm. Standard deviations (SDs) are low (0.40 to 0.55), showing good sorting. Skewness is slightly negative (−0.38 to −0.47), and kurtosis values are close to 1.0 (0.97 to 1.11), indicating mesokurtic distributions.

Finer sediments from Tenna range broadly in size: 0.66 mm (TN1) to 9.97 mm (TN3). Sorting improves in coarser samples (SD from 2.51 to 1.85), and skewness shifts from negative in TN1 (−0.64) to positive in TN3 (0.39). Kurtosis also varies (0.57 to 1.15), with TN3 again standing out for its better sorting and more peaked distribution.

#### Aso River

The Aso River samples A1, A2, and As, corresponding respectively to the first, second, and strath levels, show mean grain diameters ranging from 1.91 mm (A2) to 4.87 mm (As), with corresponding mean φ values from −0.93 to −2.28. These indicate medium to coarse gravel fractions. Sorting is poor across all samples, as indicated by high SD values (2.34 to 3.20). The distributions are positively skewed (0.20 to 0.71), with kurtosis values between 0.56 and 1.07, reflecting slightly platykurtic to mesokurtic profiles.

In the coarser fraction, mean diameters range from 13.16 mm (As) to 16.03 mm (A1), with excellent sorting (SD: 0.40 to 0.64). Skewness is negative (−0.39 to −0.50), indicating tails toward coarser material, and kurtosis remains close to 1 (0.95 to 1.11), suggesting mesokurtic distributions.

The finer fractions show much more variability, with mean diameters between 0.47 mm (A2) and 3.01 mm (As). Sorting is poor (SD: 2.37 to 2.70), and skewness transitions from negative (−0.37 in A2) to slightly positive (0.39 in As). Kurtosis values (0.69 to 0.70) suggest generally platykurtic distributions.

#### Tesino River

The Tesino River samples, TS1, TS2, and TS3, corresponding to the first, second, and third levels, show mean diameters ranging from 4.51 to 7.27 mm and a mean φ from −2.17 to −2.86. Sorting is generally poor because the SD ranges between 2.29 and 2.99, but skewness is uniformly positive ranging from 0.55 and 0.69, suggesting finer tails. Notably, TS2 displays a higher kurtosis (3.36), indicating a strongly peaked distribution, while the other samples range from 1.14 to 1.31.

The coarser fraction of the Tesino sediments is among the largest, with mean diameters from 13.64 mm (TS1) to 17.26 mm (TS3). Sorting ranges from good to excellent (SD: 0.49 to 0.82). Skewness is negative (−0.45 to −0.62), while kurtosis varies slightly (0.90 to 1.21), remaining within mesokurtic to slightly leptokurtic ranges.

The fine fraction in Tesino River varies from 1.85 mm (TS3) to 4.81 mm (TS2), with relatively poor sorting with an SD between 3.19 and 3.34. Skewness is positive (0.15 to 0.47), and kurtosis values are relatively low (0.64 to 0.66), indicating generally platykurtic distributions.

#### Grain size parameter correlation

To better understand the internal relationships among the grain size statistical parameters, a correlation analysis was performed using the Pearson correlation coefficient. This analysis considered the grain size descriptors (mean grain size, expressed both in millimeters and in φ, SD, skewness, and kurtosis) for each grain size analysis: combined, photoanalysis, and sieving. As shown in fig. S2 (see the Supplementary Materials), only correlation coefficients associated with a *P* value lower than 0.05 are reported and described, ensuring that all observed relationships are statistically meaningful.

In the combined dataset, mean grain size diameter, whether expressed in φ or millimeters, shows a strong correlation with sediment sorting and distribution shape. Finer sediments (higher φ, smaller diameter) tend to be more poorly sorted, as indicated by a positive correlation between φ and SD [correlation coefficient (*r*) ≈ 0.79] and a negative correlation between diameter and SD (*r* ≈ −0.89). Similarly, finer sediments tend to exhibit flatter distributions: Mean φ correlates negatively with kurtosis (*r* ≈ −0.73), while mean diameter shows a positive relationship (*r* ≈ 0.78) with the same parameter, suggesting that coarser samples tend to be more peaked or leptokurtic. No consistent or significant correlations were found for skewness in this dataset.

In the photoanalysis dataset, which targets the coarser grain size fractions, the relationships become even more pronounced. Increasing grain size (i.e., lower φ and higher diameter values) is associated with a moderate decrease in sorting, as shown by a negative correlation between φ and SD and a positive one between diameter and SD. Although the overall range of sorting remains relatively narrow in this coarser fraction, the trend suggests that coarser grains tend to be slightly less well sorted. In addition, the analysis reveals a moderate negative correlation between SD and skewness, indicating that more poorly sorted samples tend to be skewed toward the coarser tail. Kurtosis behaves similarly to the combined dataset: Finer samples (higher φ, lower diameter) are more peaked, while coarser ones show flatter distributions, as evidenced by the inverse correlation of kurtosis with diameter and its positive correlation with φ.

The sieving dataset, which reflects the finer sediment fraction, displays some distinct patterns. Finer sediments again tend to be more skewed toward the coarse end, as shown by a strong negative correlation between φ and skewness (*r* ≈ −0.89). This may point to the presence of coarser grains embedded within otherwise fine material, possibly reflecting secondary transport or reworking processes. At the same time, coarser fine-fraction samples (lower φ, larger diameter) tend to exhibit more peaked distributions, supported by the positive correlation between mean diameter and kurtosis. A further important trend is the negative relationship between SD and kurtosis, indicating that more poorly sorted samples display flatter, more platykurtic shapes.

Overall, the correlation analysis reveals a consistent and coherent sedimentological signal across datasets. Coarser sediments tend to be better sorted and more peaked, while finer materials are typically associated with poorer sorting, greater asymmetry, and flatter distributions.

### Fluvial terraces chronology

#### Luminescence dating

A total of eight samples was collected for postinfrared infrared stimulated luminescence (pIRIR) dating of potassium (K) feldspar, following the pIRIR_290_ single-aliquot regenerative-dose (SAR) protocol (table S2). The analyses were performed on the 100- to 120-μm or 140- to 200-μm granulometric fraction (for further details, see the Luminescence dating section in Materials and Methods). The resulting ages are listed in Table 2. Dose recovery tests (DRTs) were performed on sample FA6 on both multigrain and single-grain disks. On multigrain aliquots, the mean DRT ratio and associated SD is 0.91 ± 4% (five disks measured); on single-grain disks, the DRT ratio is 1.10 ± 43% (based on 54/300 grains measured), both after subtracting the residual dose (see figs. S3 and S4). The residual dose measured on multigrain disks for bleaching duration ranging from 30 min to 48 hours shows that remaining doses may be negligible considering the range of the equivalent doses (*D*_e_) values of 269 to 695 gray (Gy). After 30 min, a residual dose of 48 to 54 Gy was measured, which decreases up to 11 Gy after 48 hours. For this reason, no residual dose was subtracted. Overdispersion (OD) values are included between 33 ± 3 and 41 ± 2%, except for two samples for which they reach 67 ± 7% (FA3) and 77 ± 9% (FTT11) (fig. S5).

**Table 2.** Ages obtained from single-grain pIRIR_290_ dating. Equivalent dose (*D*_e_) and dose rate data, and ages obtained using the central age model [CAM; (*95*)]. The beta dose rate includes the internal (389 ± 16 μGy year^−1^) and external contributions. We assumed a potassium (K) content in the feldspars of 12.5 ± 0.5%. The cosmic dose rate was estimated using the current sample depth. The beta dose rate was calculated from uranium (U), thorium (Th), and K content measured in the sediment using laboratory gamma-ray spectrometry. The gamma dose rate was measured using a portable gamma-ray spectrometer. The 100- to 120-μm fraction was measured using the standard 150-μm hole disks, except for sample FTNN3.5 for which the 140- to 200-μm fraction was analyzed using the standard 300-μm hole disks (a few grains filled each hole). *n*, number of accepted values; *N*, number of grains measured.

River	Level	Sample #	*n*/*N*	OD (%)	*D*_e_ (Gy)	Dose rate (μGy year^−1^)					
						Alpha	Beta	Gamma	Cosmic	Total	Age (ka)
Tenna	1st	FTNN3.5	49/400	8 ± 4	456 ± 26	35 ± 7	1,187 ± 27	267 ± 6	183 ± 20	1,673 ± 28	273 ± 26
	1st	FTNN3.2	81/500	36 ± 3	695 ± 30	48 ± 9	1,540 ± 26	400 ± 13	112 ± 20	2,100 ± 30	331 ± 33
	2nd	FTNN3.4	153/500	36 ± 2	281 ± 9	67 ± 12	1,361 ± 23	263 ± 6	150 ± 20	1,842 ± 27	153 ± 14
	2nd	FTNN3.3	99/500	33 ± 3	468 ± 17	75 ± 13	1,432 ± 23	594 ± 18	160 ± 20	2,261 ± 32	207 ± 19
Aso	1st	FA3	60/500	67 ± 7	622 ± 56	45 ± 8	1,125 ± 21	433 ± 10	160 ± 20	1,763 ± 25	353 ± 42
	Strath	FA6	138/500	37 ± 4	333 ± 17	48 ± 9	1,237 ± 23	148 ± 7	207 ± 20	1,640 ± 25	202 ± 21
	2nd	FA1b	138/500	41 ± 2	269 ± 9	81 ± 14	1,511 ± 24	331 ± 8	148 ± 20	2,071 ± 30	130 ± 13
Tesino	1st	FTT11	41/500	77 ± 9	601 ± 70	82 ± 15	1,495 ± 23	669 ± 16	173 ± 20	2,419 ± 32	248 ± 35

Two samples were collected and dated from the Tenna River terrace 1 (TN1), one from a first-level deposit located 45 m above the thalweg (FTNN3.2: 331 ± 33 ka) and the second from an alluvial fan near the drainage divide (FTNN3.5: 273 ± 26 ka). The good development of the second-level terrace (TN2) allowed the sampling of both the top and the bottom of the deposit (FTNN3.4 and FTNN3.3), giving respectively an age of 153 ± 14 and 207 ± 19 ka.

In the first-level terrace of the Aso River (A1) one sample (FA3) was collected from the fine-grained portion at the top of the outcrop, obtaining an age of 353 ± 42 ka. The second-level terrace (A2) gave an age of 130 ± 13 ka. Last, from the strath terrace deposit identified on the field (As), an age of 202 ± 21 ka was obtained.

From the Tesino River, the first-level terrace has been sampled (FTT11) at the top. An age of 248 ± 35 ka was obtained, adding new chronological data to the ages already reported in (*67*) for the Tesino River.

#### Radiocarbon dating

From the third-level terrace of the Tenna River (TN3), the sample FTNT4 was collected for radiocarbon analyses (Fig. 2). The obtained calibrated age was 39.640 ± 0.407 ka (for further details, see table S3 in the Supplementary Materials).

## DISCUSSION

### Climatic influence on terrace formation

**Fig. 6.**
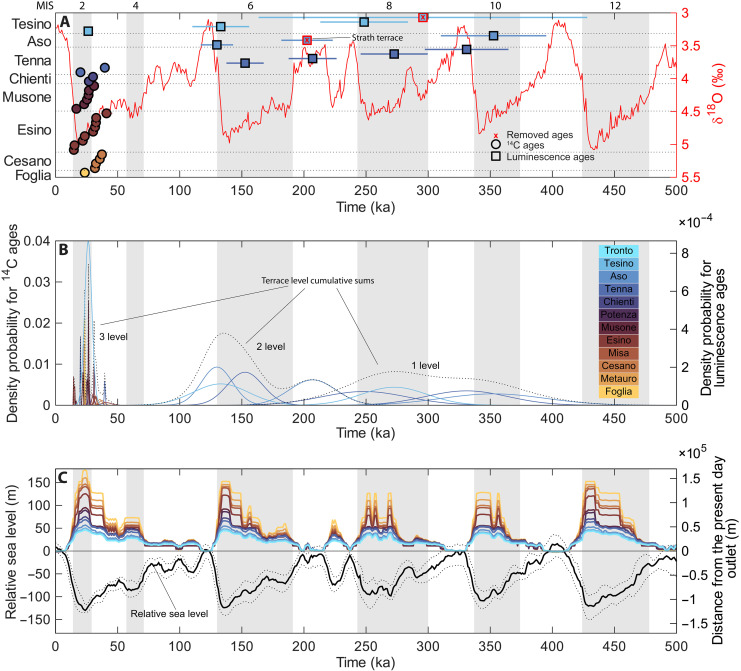
Overall available ages and their density probabilities plotted against the δ^18^O, global eustatic curves, and the planar shoreline distance from the present day outlet of the last 500 ka. From the top: (**A**) All the available ages for fluvial terraces of the peri-Adriatic belt (for more details, see table S1) plotted on the δ^18^O curve of the last 500 ka (*111*); (**B**) normal distribution probability densities of the available ages distinguished by method and the cumulative sums relative the terrace levels; and (**C**) global eustatic curve updated from (*112*) and planar shoreline distance to the present day outlet of the peri-Adriatic rivers during the last 500 ka. Circled in red are the ages that were not considered in the cumulative sums: (i) the strath terrace in which the sedimentation phase is neglectable and (ii) the first-level age of the Tesino River with ~45% relative error.

From the analysis of the peri-Adriatic belt rivers, including this study’s rivers integrated with previously published data (table S1), it can be observed that climate variations have a fundamental role in the sedimentation of the terrace deposits. As shown in Fig. 6, the correspondence between the alluvial sedimentation and the cold phases strengthens toward the present with highest correlation during marine isotope stage (MIS) 2. Here, it can be observed that the sedimentation begins within the cooling phase at the end of the previous interglacial, and it finishes at the end of the glacial phase. Secondarily, MIS 6 also shows a good relationship, while MIS 8 and MIS 10 show weaker relationships.

This climate-sedimentation link was already proposed on a morphostratigraphic basis by several authors [e.g., (*37*, *68*)], but earlier validations were largely restricted to terraces correlated with MIS 2 (see table S1). Delchiaro *et al.* (*67*) extended the same interpretation also to the older terraces of Tesino River, but the chronological constraints available at the time were not sufficient to produce a robust regional interpretation.

Figure 6C illustrates the nearest planar distance between the actual outlet of the peri-Adriatic rivers and the isobath corresponding to the relative sea level over time. It reveals that the maximum distance reached during cold phases is higher in the north (Foglia River up to 150 km) and lower to the south (up 40 km for Tronto River). Overall, the peri-Adriatic belt was positioned well inland to the coastline, discounting the impact of glacioeustatic base level falls for terrace genesis and supporting their climate-driven formation. This is attributed to the low-gradient bathymetry of the Adriatic Sea (*71*), which caused notable coastline progradation during sea level lowstands (*72*).

The dynamics that link fluvial deposition and terrace formation to cold phases is commonly explained by the effect of extreme climatic conditions on sediment production in river basins (*73*). These conditions enhance mechanical weathering, affecting the slopes with reduced vegetation cover and leading to major sediment production that is transported to the valley floor by mass wasting and runoff (*9*, *11*, *13*). However, the potential stratigraphic incompleteness and the increasing likelihood of hiatuses due to infrequent events governed by heavy-tailed distributions of water discharge and sediment flux should be accounted for toward longer integration timescales (*22*–*24*). This is particularly relevant for the luminescence ages that overlap across MIS 8 to 10. Despite the new chronological dataset presented here, the number of absolute age constraints remains insufficient to fully validate the long-term climatic interpretation. Furthermore, during the Middle Pleistocene, the drainage basins were smaller and less mature, implying shorter sediment routing distances and response times, which may also have influenced terrace development.

From a sedimentological perspective, the fluvial terraces deposits of the Tenna, Aso, and Tesino rivers present facies associations [sensu (*70*)] that are quite consistent with the typology of deposits identified by Nesci *et al.* (*68*) in the peri-Adriatic belt basins. In particular, the typical sequence identified is composed of alluvial fan deposits at the top of braided river deposits. This stratigraphic transition is a natural sedimentologic sequence for fill terraces that have been abandoned by the main river channel and then modified by hillslopes and tributaries (*7*). This abandonment was attributed by Nesci *et al.* (*74*) to temperature lowering during the final part of the glacial stages.

Although the grain size distribution in the fluvial deposits is heterogeneous and therefore limits spatial representativity, this issue was mitigated through a combined methodological approach (photoanalysis and laboratory sieving) that provided a more comprehensive characterization. Overall, the sedimentological observations are consistent with the grain size analysis results. In the oldest terraces (levels 1), the sediments are finer, poorly sorted, and characterized by flatter (platykurtic) distributions, indicating lower-energy depositional conditions and possible postdepositional reworking. In contrast, in the more recent levels (level 3), coarser grain sizes are observed, along with better sorting and more peaked (leptokurtic) distributions, consistent with higher-energy and more rapid depositional phases, probably linked to climatic transitions toward colder periods. This trend is particularly evident in the Tenna and Tesino basins, where the vertical evolution of the terraces shows a progressive improvement in sedimentological characteristics with decreasing age. Statistical correlations among the granulometric parameters support this model: Coarser sediments tend to be better sorted and exhibit more peaked (leptokurtic) distributions, whereas finer materials display poorer sorting, higher skewness, and flatter distributions.

### Tectonic signal from terrace fill deposits

One of the questions addressed in this work concerns the possibility of extracting tectonic signals from terrace deposits of the fill type [sensu (*8*)]. Laterally and vertically mobile rivers rework valley floor materials over time (*75*), and the multiphase activity leads to the insetting of terrace units and their overlapping. For this reason, in the case of fill terraces, it can be challenging to identify a singular equivalent point in the sedimentological record to connect between successions. This issue was highlighted by Pederson *et al.* (*76*) that proposed a possible solution into the precise and accurate chronological assessment of both the basal and upper portion of these deposits. This provides a more reliable datum regarding the onset and the end of sedimentation, allowing to extract reliable long-term fluvial incision rates and interpret them as uplift rates. In particular, to derive the tectonic uplift rate, the elevation of the strath basal surface with respect to the modern channel elevation is considered.

**Fig. 7.**
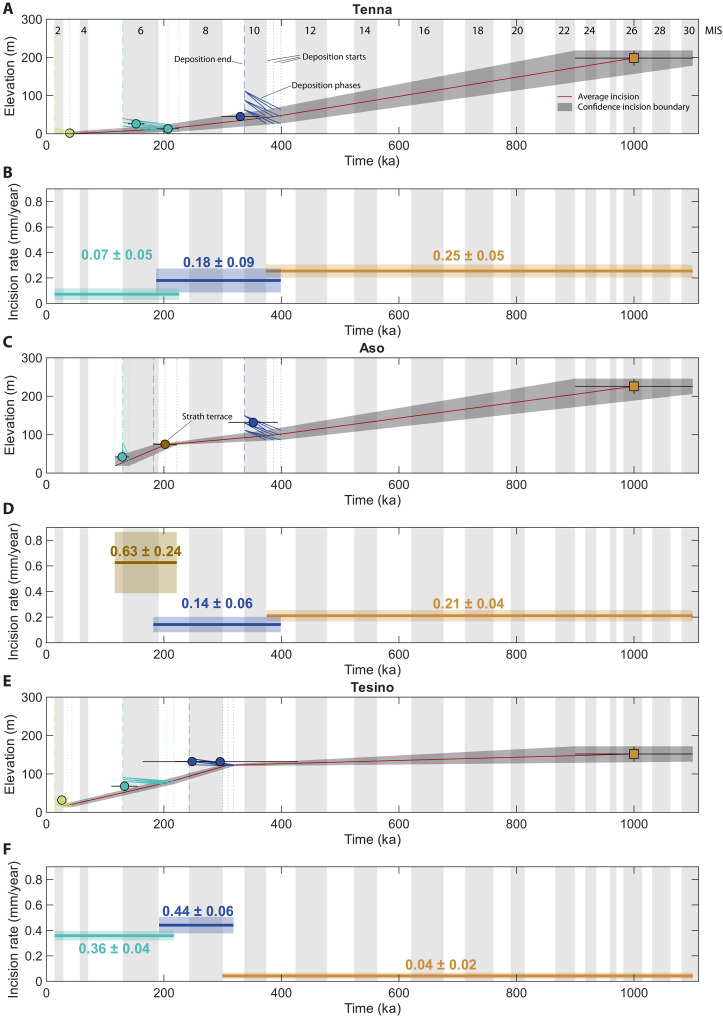
Fluvial incision rates computed for the three study basins. Thalweg elevation through time, highlighting the deposition-incision cycles related to the terrace levels upon which uplift rates are estimated related to the Tenna (**A** and **B**), Aso (**C** and **D**), and Tesino (**E** and **F**) rivers. The age-elevation coordinates related to the luminescence and radiocarbon samples are also reported.

In this study, the methodology proposed by Pederson *et al.* (*76*) for fill terraces appeared to be reasonably applicable. The ages of the bottom and of the top of each terrace deposit were taken into account; however, the observations related to the climatic influence on terrace formation, observed for MIS 2 and 6, provided a solution to complement the methodology when the chronological constraints were missing. In detail, it was assumed that the beginning of deposition began within the cooling phase preceding each glacial MIS. As shown in Fig. 7, the base of the third-level terrace of the Tenna River (TN3) and the second-level terrace of the Tenna (TN2) and Aso (A2) rivers were successfully dated and used for modeling. Because for other terrace deposits (TS1, TS2, TS3, A1, and TN1), a precise chronological constraint from the basal strath surface was not obtained, it was assumed that the beginning of deposition began with the respective start of the cooling phase preceding each glacial MIS. An exception from this approach is represented by the strath terrace deposit identified in the Aso River (As). The interpretation here is simplified by the fact that it captures a “snapshot” of the valley floor level at the time of its deposition (202.1 ± 20.5 ka), thereby simplifying its interpretation. The floodplain deposits were not considered in this study to avoid potential biases related to the Sadler effect, which leads to an overestimation of incision rates, as the floodplain does not yet represent a complete sedimentary record.

To quantify model uncertainty, we incorporated in the model the start, middle, and end of each cooling phase, as well as the elevation of the strath surface and its associated error for each terrace level. In addition, to extend the modeled history to Early Pleistocene, we also took into account the base of Fermo Fm. considering a total thickness of 40 m with an age 1 ± 0.2 Ma (*69*). The age modeling then estimated all possible age-elevation combinations, accounting for uncertainties in consecutive strath surface levels, and returned mean uplift rates along with their SDs (Fig. 7). From the age-elevation models, we reconstructed uplift-rate histories for the three river basins.

For the Tenna River, uplift proceeded at 0.25 ± 0.05 mm year^−1^ from 1 ± 0.2 Ma to 386.5 ± 12.5 ka (cooling phase of MIS 11). It then decreased to 0.18 ± 0.09 mm year^−1^ between 386.5 ± 12.5 and 206.9 ± 19 ka (age of the base of the second-level terrace). A further decrease to 0.07 ± 0.05 mm year^−1^ occurred from 206.9 ± 19 to 39.6 ± 0.4 ka (age of the base of the third-level terrace).

The Aso River shows a similar early history, with an uplift rate of 0.21 ± 0.04 mm year^−1^ from 1 ± 0.2 Ma to 386.5 ± 12.5 ka (MIS 11 cooling phase). The rate declined to 0.14 ± 0.06 mm year^−1^ between 386.5 ± 12.5 and 202.1 ± 20.5 ka (strath-level terrace). It then increased sharply to 0.63 ± 0.24 mm year^−1^ from 202.1 ± 20.5 to 129 ± 12 ka (base of the second-level terrace).

The Tesino River experienced a low uplift rate of 0.04 ± 0.02 mm year^−1^ from 1 ± 0.2 Ma to 386.5 ± 12.5 ka (MIS 11 cooling phase). This rate rose to 0.44 ± 0.06 mm year^−1^ between 386.5 ± 12.5 and 204 ± 13 ka (MIS 7 cooling phase), before decreasing slightly to 0.36 ± 0.04 mm year^−1^ from 204 ± 13 to 36 ± 7 ka (MIS 3 cooling phase).

In general, the uplift rates obtained in this study are consistent with earlier findings for the last 500 ka, allowing to extend the age model histories until Early Pleistocene. In the Umbria-Marche Apennines, uplift rates have been estimated ranging between 0.3 and 0.5 mm year^−1^ (*36*, *37*, *40*, *63*, *64*, *67*, *77*), increasing toward the orogenic axis.

### Implications on the peri-Adriatic belt Quaternary evolution

By providing new chronological constraints, this study strengthens the existing dataset of terrace ages in the peri-Adriatic belt and allows tectonic and climatic signals to be more isolated within the stratigraphic record of fluvial fill terraces. Consequently, it is now possible to infer the landscape evolution of the region during the Quaternary.

**Fig. 8.**
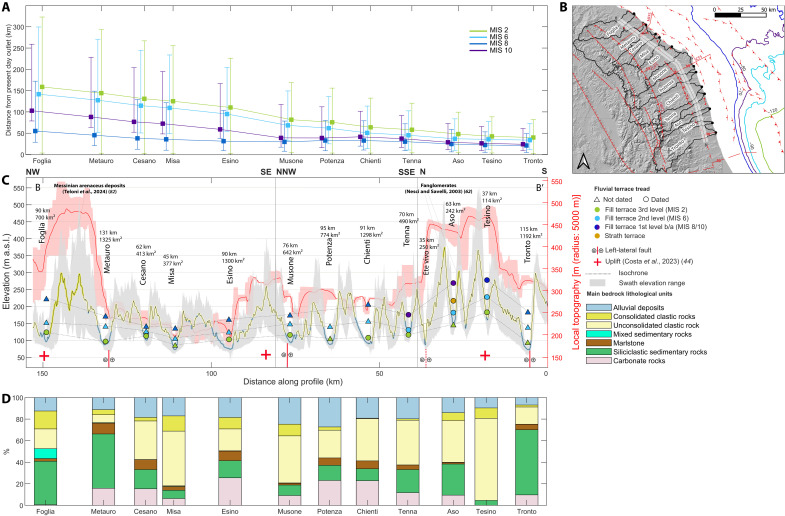
Comprehensive schematics of Marche region with the data collected in this study. The distance of each catchment’s outlet during lowstands from the sea line (**A**) and the corresponding isobaths (**B**) are reported. In (**C**), topographic and local relief swath profiles, oriented perpendicular to the trunk valleys, display the height distribution of terraces throughout the Apennines peri-Adriatic belt. The swath profile trace is indicated in Fig. 1 (B-B′), with a width of 5 km. Uplift indicated from (*44*). Reported faults in red from the Individual Seismogenic Sources from the DISS catalog [3.3.0 (*109*)]. In (**D**), the percentage of the different lithologies inside the basins from the spring to the outlet is shown.

Figure 8C shows the main terrace staircases of the peri-Adriatic belt, including the studied basins, projected along a topographic transect intended as a swath profile with a width of 5 km traced parallel to the coastline, 10 km inland (for the precise location, see Fig. 1, transect B-B′). The distribution of the terrace levels is not homogeneous: It can be observed that the same terrace levels are distributed at different heights among fluvial valleys. The Foglia, Tesino, and Aso river terraces are located at higher elevation in comparison with the same levels of the other river valleys. This positive anomaly is mirrored also by the local topography that records the highest values along the transect in their correspondence, reaching values around 500 m. In this regard, the lithologies that characterize the topographic transect in Fig. 8C are mostly unconsolidated clastic rocks and additionally consolidated clastic rocks outcropping in correspondence to the local topography anomalies along the interfluves between Foglia and Metauro rivers and Ete Vivo and Tronto rivers. They can be distinguished as terrigenous deposits attributable to the Messinian in the north, while in the south, they are interpreted as coastal fans or fanglomerates (Fig. 8C) (*62*). However, the thickness of the consolidated clastic rocks is not proportional to the local topography anomalies, and consequently, it can be assumed that they are not totally related to each other. A factor that may nonetheless influence the elevation at which terraces occur is the erodibility of the lithologies present within the catchments. A more erodible bedrock will result in greater sediment supply to the hydrological system (*78*). In this regard, the analysis of the lithological percentages within the basins (Fig. 8D) shows that, excluding the alluvial deposits, the outcropping lithologies generally exhibit similar erodibility. Nonetheless, the Tesino and Foglia catchments do not include carbonate lithologies within their extent. Looking instead at the percentage of alluvial deposits within the basins, the highest values are observed in the central part of the transect, with percentages of around 30%. Conversely, the lowest percentages are found at the ends of the transect, between the Aso and Tronto rivers in the south, and the Foglia and Metauro rivers in the north. This does not appear to be correlated with the drainage area associated with each basin (*79*). For instance, the Esino basin is twice the size of the Musone basin, yet their percentages of alluvial deposits are comparable. A similar behavior can be observed between the Tenna and Chienti and between the Tesino and Tronto basins.

These observations indicate that the terrace staircases’ positive elevation anomalies primarily reflect tectonic processes. The differential uplift recorded along the terraces is consistent with previous estimates for the last 500 ka (*36*, *37*, *40*, *63*, *64*, *67*, *77*), but our dataset allows a more detailed analysis of recent Quaternary deformation. This also enriches other studies’ interpretations, which discuss geodynamic processes on a broader timescale, typically focusing on the past 3 Ma [e.g., (*64*)]. In particular, these studies found that a rock uplift pulse started around 3.0 to 2.5 Ma, coinciding with the onset of extension in the Apennines. Then, a renewed increase in rock uplift rates occurred after the Middle Pleistocene along the Adriatic coast, coeval with recent uplift acceleration along the eastern coast of southern Italy (*80*). While the rapid rock uplift pulse between 2.5 and 1.5 Ma may be associated with propagation of slab break-off, this mechanism does not explain the accelerated Middle Pleistocene to present-day rock uplift. As a result, the greater uplift observed in the river terrace staircases of the Foglia, Metauro, Aso, and Tesino rivers suggests a spatially nonuniform uplift pattern.

On one hand, the different elevation of terrace levels can be attributed to differential uplift to a tectonic configuration of the region characterized by crustal blocks disarticulated by strike-slip faults, such as MFFS (*81*) and SCFS (*58*), hypothesis that is also consistent with (*43*, *44*) and (*82*). On the other hand, an alternative explanation supports a nonuniform growth of anticlines striking north-northwest-south-southeast, parallel to the coastal chain (*56*). Differential uplift of terrace staircases results then from along-strike variations in anticline growth rather than strike-slip motion. In this sense, strike-slip faults may have limited vertical components, but they are unlikely to be the main driver of the differential uplift observed.

Furthermore, most earthquakes and mainshocks in the region, including the latest offshore *M*_w_ 5.5 Fano-Pesaro earthquake of 2022, are contractional and roughly parallel to the coast and anticline axes (*52*, *53*, *56*). Minor strike-slip events with anti-Apennine (northeast-southwest) direction (*35*, *59*) would represent secondary ruptures affecting small fault segments and do not reflect regional deformation. In this framework, the SCFS and MFFS may function as transfer zones accommodating differential anticline growth.

Nevertheless, further investigations are needed to test these hypotheses. The main limitations of this study lie in the relatively small number of samples from the oldest terraces, despite obtaining seven dates for first- and second-level terraces. Ideally, sampling the oldest terrace levels across multiple rivers in the peri-Adriatic belt, as well as collecting additional samples within the same deposit, would improve age accuracy. Moreover, in the Pederson model application, we link fluvial sedimentation to cold phases starting from the cooling phase of the previous warm period. This represents a simplification of more complex natural processes (e.g., cut-and-fill dynamics). Additional detailed geomorphic analysis of fault scarps and anticline axes and high-resolution geophysical imaging of subsurface structures could also provide quantitative constraints on uplift rates and the relative contribution of faults versus anticlines to landscape deformation.

## MATERIALS AND METHODS

### Experimental design

The study started with a first phase dedicated to the mapping of the fluvial terraces’ features, including the upper tread and the associated alluvial deposits. The former features were identified using a semiautomatic mapping remote technique, while for the latter, the Quaternary deposits of the geological map 1:10,000 (available at www.regione.marche.it/Regione-Utile/Paesaggio-Territorio-Urbanistica/Cartografia/Repertorio/Cartageologicaregionale10000) were taken into consideration. After this preliminary analysis, the tread surfaces together with the associated alluvial deposits were checked in the field. In addition, the fieldwork was instrumental for the collection of the samples necessary to pursue the sedimentological and the chronological characterization of the deposits using luminescence dating techniques.

In this regard, the sedimentological analysis aimed to assess qualitatively the different depositional facies and to differentiate the strictly alluvial deposits from the colluvial ones. It was then complemented by a grain size analysis at the exposure scale of the sampling outcrops. The statistical grain size distribution results helped to reconstruct the depositional flow conditions associated with each terrace level.

### Semiautomatic mapping of terrace treads

The procedure of terrace tread semiautomatic mapping was performed using freely available high-resolution light detection and ranging DTMs of the three basins with a vertical accuracy of ±15 cm, a horizontal accuracy of ±30 cm, and a spatial resolution of 1 m, acquired from Ministero dell’Ambiente e Sicurezza Energetica during 2008 to 2010 (https://sim.mase.gov.it/portalediaccesso/mappe/#/viewer/new). First, the Relative Elevation Model [REM; or height above river raster (*83*)] has been extracted using the QGIS software. By applying the inverse distance weighting processing on the river centerline point vector, the weighted average of elevations within a search radius of each grid cell generated an interpolated plane with elevations relative to the river centerline. This is subtracted to the original DTM, ending up with a raster where the elevations trend higher, moving away from the thalweg. The relative elevation of the different deposits is then used in the next analysis to operate a preliminary classification into different terrace levels.

The second step of the process uses the MATLAB-based TopoToolbox (*84*) to operate the semiautomatic extraction of the possible terrace upper surfaces. This second analysis leverages the fluvial terrace characteristic slope (*SLP*) and roughness (*RG*), enabling their identification. The base of this technique is the surface classification model (SCM) developed by Bowles and Cowgill (*85*) for the identification of potential marine terraces, which makes the most of the topography of these landforms. In particular, the slope and the roughness of the landscape are combined in the SCM index to identify the areas characterized by flat to gently inclined and smooth surfaces, semiautomatically detected as follows

$$\text{SCM}=\left(\frac{\mathrm{SLP}<\text{SLPr}}{\text{SLPr}}\cdot 0.5\right)+\left(\frac{\mathrm{RG}<\mathrm{RGr}}{\mathrm{RGr}}\cdot 0.5\right)$$ (1)

where *SLPr* and *RGr* are the specific slope and roughness cut-offs, respectively.

In detail, from the DTM resampled at 5 m, a slope map was generated and then clipped by setting to null all slopes > 5° (*SLPr*) (Eq. 1). Then, the resulting slope values were normalized by *SLPr*. At the same time, the surface roughness, intended as the SD of the slope, has been computed. The roughness cut-off (*RGr*) was set at the 0.9 quantile, which incorporates 90% of the roughness values, allowing to remove outliers. For the Tesino River, it was set at 2.46, for the Aso at 2.06, and for the Tenna at 1.42. The resulting roughness values were then normalized by *RGr*. Afterward, the resulting SCM values have been filtered below the 0.9 quantile. This was equal to 0.52 for the Tesino, 0.52 for the Aso, and 0.55 for the Tenna River. The areas with SCM values near to 0 indicate a possible terrace feature or the alluvial plain, characterized by both low slope and low roughness. Then, the patches obtained from the SCM were intersected with the REM elevation values. The resulting tread surfaces were then classified on the basis of the frequency distribution of their relative elevation values. Ultimately, the final classified patches have been cross-checked in the field together with the associated alluviums obtained from the geological map 1:10,000 (available at www.regione.marche.it/Regione-Utile/Paesaggio-Territorio-Urbanistica/Cartografia/Repertorio/Cartageologicaregionale10000).

### Sedimentological, grain size, and statistical analyses

The general sedimentological characterization of the terrace deposits was conducted following the Miall classification (*70*), which was adopted for the description of the different facies present in the terraces’ exposures (see Figs. 2 to 4).

In addition, on the sampling outcrops, grain size analyses were performed. In particular, given the heterogeneous nature of grain size distribution in fluvial deposits, a combined methodological approach was used to ensure comprehensive characterization. This included (i) photoanalysis conducted with the support of BASEGRAIN 2.2, a MATLAB-based automatic object detection software tool (*86*), and (ii) laboratory sieving of the sediment samples.

The photosieving technique takes pictures taken orthogonally to the outcrops and imports them in the BASEGRAIN software, which uses a grayscale threshold approach for grain identification. First, the dimensions of the grains are calibrated with a scale that is manually determined by measuring the ruler object inside the picture. Then, the interstices are separated from the grain areas, and the grain sizes are determined. This is made possible by selecting a small rectangular area to calibrate the image identification parameters, where the filtering thresholds are progressively adjusted for the most optimal identification of the particle edges and surfaces. If the grains still cannot be correctly identified by the software, then in the postprocessing phase, it is possible to merge and split the edges to follow the correct shape of the grains. After the postprocessing, the results are saved as an Excel spreadsheet file, containing grain size curves, grain size statistics, and the properties of each detected grain. The photosieving technique was conducted in the size range between 1500 up to 9.5 mm.

The sieve tests were conducted at the geotechnical laboratory at Sapienza University of Rome by mechanical separation following the American Society for Testing and Materials (ASTM) D422-63 (*87*) recommendations. The sieving tests were performed in the size range between 75 μm (held up by the sieve no. 200) and 9.5 mm (held up by the sieve no. 3/8).

Grain size cumulative curves were generated separately for both the photoanalysis and sieving analysis results and for the combined datasets. A statistical analysis was then performed on the grain size analysis results following the parameters of Folk and Ward (*88*). From the cumulative curves, the following grain-size parameters were obtained

$$\text{Mean size}=\frac{\varphi_{16}+\varphi_{50}+\varphi_{84}}{3}$$ (2)

$$\text{Standard deviation}=\frac{\varphi_{84}-\varphi_{16}}{4}+\frac{\varphi_{95}-\varphi_{5}}{6.6}$$ (3)

$$\text{Skewness}=\frac{\varphi_{16}+\varphi_{84}-2\varphi_{50}}{2\left(\varphi_{84}-\varphi_{16}\right)}+\frac{\varphi_{5}+\varphi_{95}-2\varphi_{50}}{2\left(\varphi_{95}-\varphi_{5}\right)}$$ (4)

$$\text{Kurtosis}=\frac{\varphi_{95}-\varphi_{5}}{2.44\left(\varphi_{75}-\varphi_{25}\right)}$$ (5)

where the subscript for ϕ indicates the cumulative percentile values. The size is expressed both in millimeters and in ϕ, where ϕ = −log_2_ (size in millimeters).

The mean size expresses the average width of the grains. The SD defines the variability or spread of a size class, and it is inversely proportional to the degree of sorting. The skewness expresses the degree of symmetry in the distribution of the grains at the various dimensions. When the distribution is perfectly symmetric, the skewness is equal to 0, and positive values are obtained when the slope of the cumulative curve toward the finer fractions is lower than the slope toward coarser grain-size fractions, while it is negative in the opposite case. Last, the kurtosis is the ratio between the slope of the cumulative curve in its central part and the slope toward its ends. It therefore expresses the prevalence of the intermediate grain size fractions with respect to the extreme ones.

### Luminescence dating

Geochronological analyses using luminescence protocol [pIRIR_290_ (*89*); see table S2] applied on potassium feldspars have been performed to date the timing of sediment deposition within the fill terraces. This method allows dating the last exposure to light of the sediment before deposition. It is based on the capacity of natural minerals to record a dose of ionizing rays coming from the sedimentary environment of the sample and the cosmic rays. The dose stored by the feldspar is called the equivalent dose (*D*_e_) and can be released by stimulating the sample with light (*90*). Infrared stimulated luminescence (IRSL) dating leverages the propriety of feldspar grains contained in the sediment to produce blue emissions when stimulated using infrared light (*91*). The method relies on the condition that the feldspar IRSL signal was completely bleached by sunlight before deposition; otherwise, the presence of a residual dose can cause age overestimation. To convert it into an age, the *D*_e_ is divided by the annual dose received by the sample (dose rate):

$$\text{Age}\;(\text{ka})=\frac{\text{Equivalent dose}\;\left(\text{Gy}\right)}{\text{Dose rate}\;\left(\frac{\text{Gy}}{\text{ka}}\right)}$$ (6)

The sediment samples were collected from fresh sections using opaque metal tubes to prevent interaction with light. Possible reworking after the sample removal from the outcrop has been prevented by pushing the tubes into the sediment and sealing them tightly at both ends with fitting lids. In-situ gamma spectrometry measurements were conducted using a portable gamma-ray spectrometer connected to a LaBr probe (Inspector 1000, Canberra) inserted in the excavations left from the sample, extended to reach 30 cm deep inside the section. The in-situ gamma dose rates were obtained following the “threshold technique” (*92*). The samples were then prepared and analyzed at the luminescence laboratory Archéosciences Bordeaux (Université Bordeaux Montaigne, France). All the preparations of the samples were conducted in a dark room, under subdued red and orange light. The sediment at the two ends of the tube, exposed to light during sampling, was used for water content measurements, and gamma spectrometry analyses performed in the laboratory using a spectrometer with broad energy germanium (Ge) detector to determine the U, Th, and K content (see table S4) and derive the alpha and beta dose rate. The sediment from the center of the tube was kept for *D*_e_ determination.

The samples were wet sieved and chemically treated to eliminate organic matter [using hydrogen peroxide (H_2_O_2_); 30%] and carbonates [using hydrochloric acid (HCl) 10%]. Densimetric separation was performed with heavy liquid sodium polytungstate with a density of 2.72 g cm^−3^ for the heavy minerals and then 2.62 g cm^−3^ for the quartz, and lastly, the K-feldspar fraction was isolated by densimetric separation at density 2.58 g cm^−3^.

The quartz signal being saturated, the pIRIR_290_ signal of the feldspars was used for *D*_e_ determination following the protocol by Thiel *et al.* (*89*) presented in table S2. Single-grain analyses (*93*) were executed on the most dominant granulometric fraction (100 to 120 μm or 140 to 200 μm; for further details, see table S5) with a TL/IRSL DA-20 Risø reader, and the signal was detected using a combination of optical filters. The luminescence data were processed using Analyst v. 4.56 (*94*): The single-grain signal was integrated using the first 0.05 s, and the background was subtracted from the last 0.27 s. The fit to the dose response curve was exponential + linear. The following criteria were applied for the data selection: a recycling ratio limit of 10%, a recuperation <5% of the natural signal, a maximum test dose error of 10%, and a test dose signal >3 sigma above background.

The central age model [CAM; (*95*)] was used to calculate the equivalent dose for each sample. The CAM model takes OD into account when determining the weighted mean and its standard error, and it also provides an estimate of its OD (*96*). The pIRIR_290_ signal tends to bleach slowly (*97*), making it possible for a residual dose to still be present even after prolonged light exposure (*98*). To investigate how the bleaching time influences the residual dose, the multigrain aliquots of FA6 sample were bleached in a solar simulator (Hönle 500) for periods of 4, 24, 48, and 120 hours (three aliquots each; for the results, see figs. S3 and S4). For the multigrain analyses, the signal was integrated using the first 5 s, and the background was subtracted from the last 50 s. DRTs were conducted on both multi- and single grain. Multigrain disks (*n* = 10) were bleached in a solar simulator (Hönle 500) for ~4 hours and 30 min: Five disks were used to measure the residual dose and the five remaining ones to perform the DRT (added dose of 293 Gy). For the single-grain analyses, 600 grains were bleached in a solar simulator for 4 hours: Three hundred grains were analyzed to measure the residual dose, and the DRT was conducted on the 300 remaining grains (added dose of 303 Gy). For both multi- and single-grain analyses, the DRT ratios were calculated after subtracting the residual dose (fig. S3). The dose rate was derived using the conversion factors of Guérin *et al.* (*99*), and the ages were calculated with the 1 σ error range taking into account beta absorption factors of Guérin *et al.* (*100*), alpha attenuation factors of Brennan *et al.* (*101*), and an a-value of 0.08 ± 25%. The cosmic rate was estimated using the current depth of the samples following (*102*). A water content of 10% was assumed.

### Radiocarbon dating

Radiocarbon dating was used to constrain the age of the Tenna third-level river terrace (sample FTNT4). The charcoal and plant matter were sampled and dated using accelerator mass spectrometry. The analyses were conducted at the Centre for Isotopic Research for Cultural and Environmental Heritage at the University of Campania “Luigi Vanvitelli.”

Here, the sediments were initially subjected to preliminary sieving with a mesh (Ø 60 mm) to separate them from any other coarse extraneous components (such as plant remains, lithic fragments, etc.). Subsequently, the sediment undergoes chemical treatment with acid digestion [3% (v/v) HCl] to remove the inorganic fraction of carbonate origin that was not eliminated by sieving. The acid treatment is performed under heating conditions (*T* = 80°C) on a hot plate to accelerate the reaction. The effervescence produced during acid digestion indicates the formation of carbon dioxide (CO_2_), resulting from the reaction of the acid with carbonates. The duration of the treatment varies (from several hours to several days, with multiple additions of acid and intermittent stirring) depending on the amount of carbonates present and is continued until effervescence ceases. Last, the sediment is rinsed with demineralized water to neutral pH and dried in an oven at 70°C overnight (*103*).

Once combusted and the CO_2_ purified in a high-vacuum cryogenic line, the gas is graphitized according to the zinc reduction method (*104*). The resulting graphite is then pressed into an aluminum cathode and loaded into a holder for measurement, together with blanks and reference standards. Measurements are performed using a 3-MV particle accelerator (*105*) located at the PoLaR (Research Laboratory Hub) of the University of Campania Luigi Vanvitelli. The measured data are processed to obtain isotopic ratios, which are then converted to radiocarbon ages and subsequently calibrated to calendar ages through a dedicated calibration procedure. Radiocarbon ages were calibrated using OxCal v4.4.4 (*106*) using the InCal20 calibration database (*107*).
