# Supplementary material for: Quaternary landscape evolution of Apennines peri-Adriatic belt: Insights into climate and tectonics from the fluvial record
Source: Sci Adv. 2026 Apr 24;12(17):eaec5376. doi: 10.1126/sciadv.aec5376 (PMC13108535; doi:10.1126/sciadv.aec5376)
Supplement: Supplementary file 1 — Supplementary Text Figs. S1 to S5 Tables S1 to S5 [file sciadv.aec5376_sm.pdf]

Science Advances
AAAS

Supplementary Materials for

Quaternary landscape evolution of Apennines peri-Adriatic belt: Insights into climate and tectonics from the fluvial record

Valeria Ruscitto *et al.*

Corresponding author: Valeria Ruscitto, valeria.ruscitto@uniroma1.it;
Michele Delchiaro, michele.delchiaro@uniroma1.it

Sci. Adv. **12**, eaec5376 (2026)
DOI: 10.1126/sciadv.aec5376

This PDF file includes:

Supplementary Text
Figs. S1 to S5
Tables S1 to S5

Supplementary Text

IRSL dating on feldspar from the Tenna, Aso and Tesino River (Italy)

Eight sediment samples from the Tenna, Aso and Tesino Rivers were dated using Infrared Stimulated Luminescence (IRSL) of feldspar.
For equivalent dose (D_e) determination the $pIRIR_{290}$ signal of the feldspars was used following the protocol of (89) presented in Table S2.
The U, Th, and K contents are presented in Table S4, together with the in-situ gamma dose rates.
Dose Recovery Tests (DRT; Fig. S3 and S4) were performed on both multigrains and single grains disks. On multigrain aliquots, the mean DRT ratio and associated standard deviation is 0.91 ± 4% (five disks measured); on single grain disks, the DRT ratio is 1.10 ± 43% (based on 54/300 grains measured), both after subtracting the residual dose.

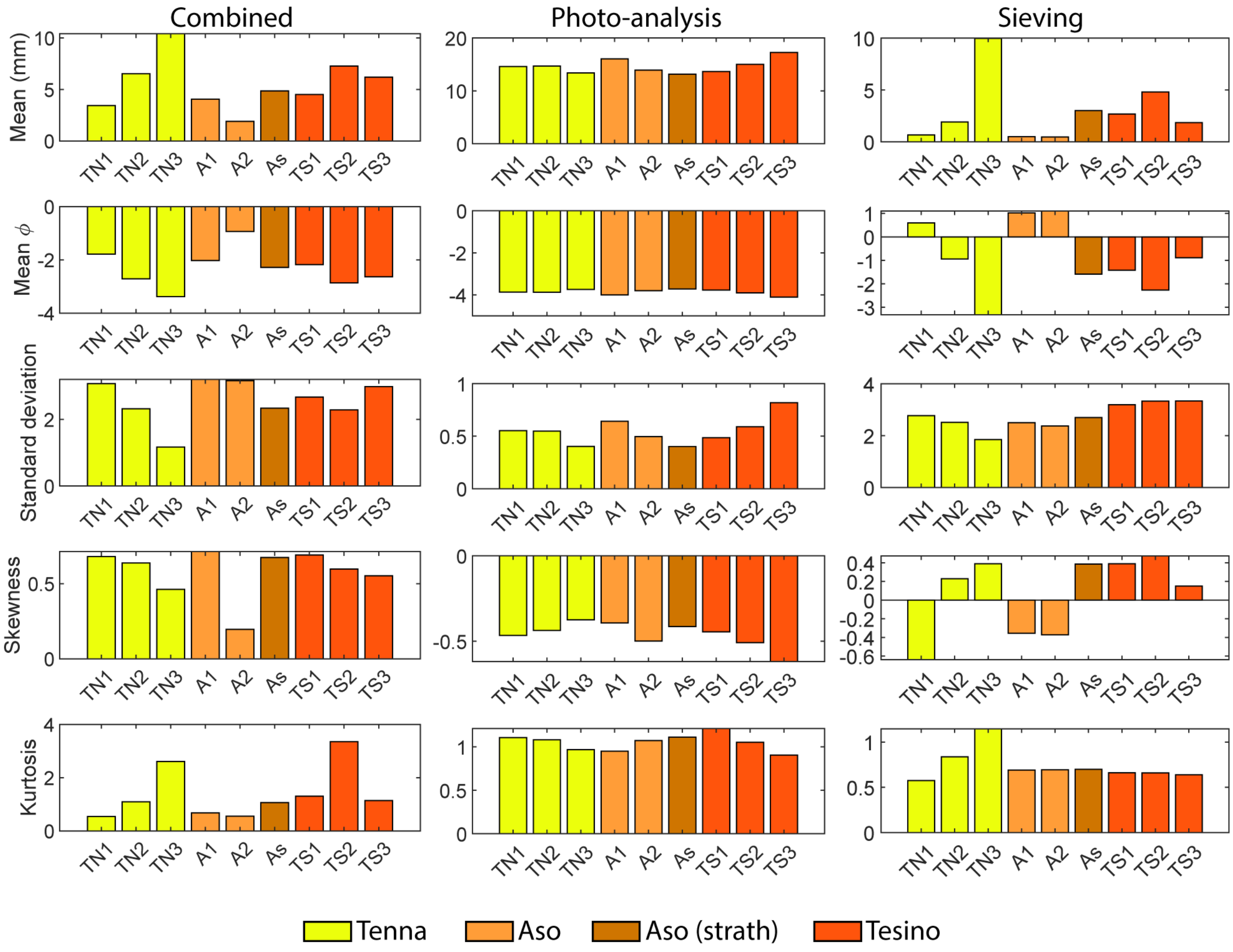

Fig. S1.
Grain-size analyses results of the different rivers' levels carried out in three modalities: combined, photo-analysis and sieving.

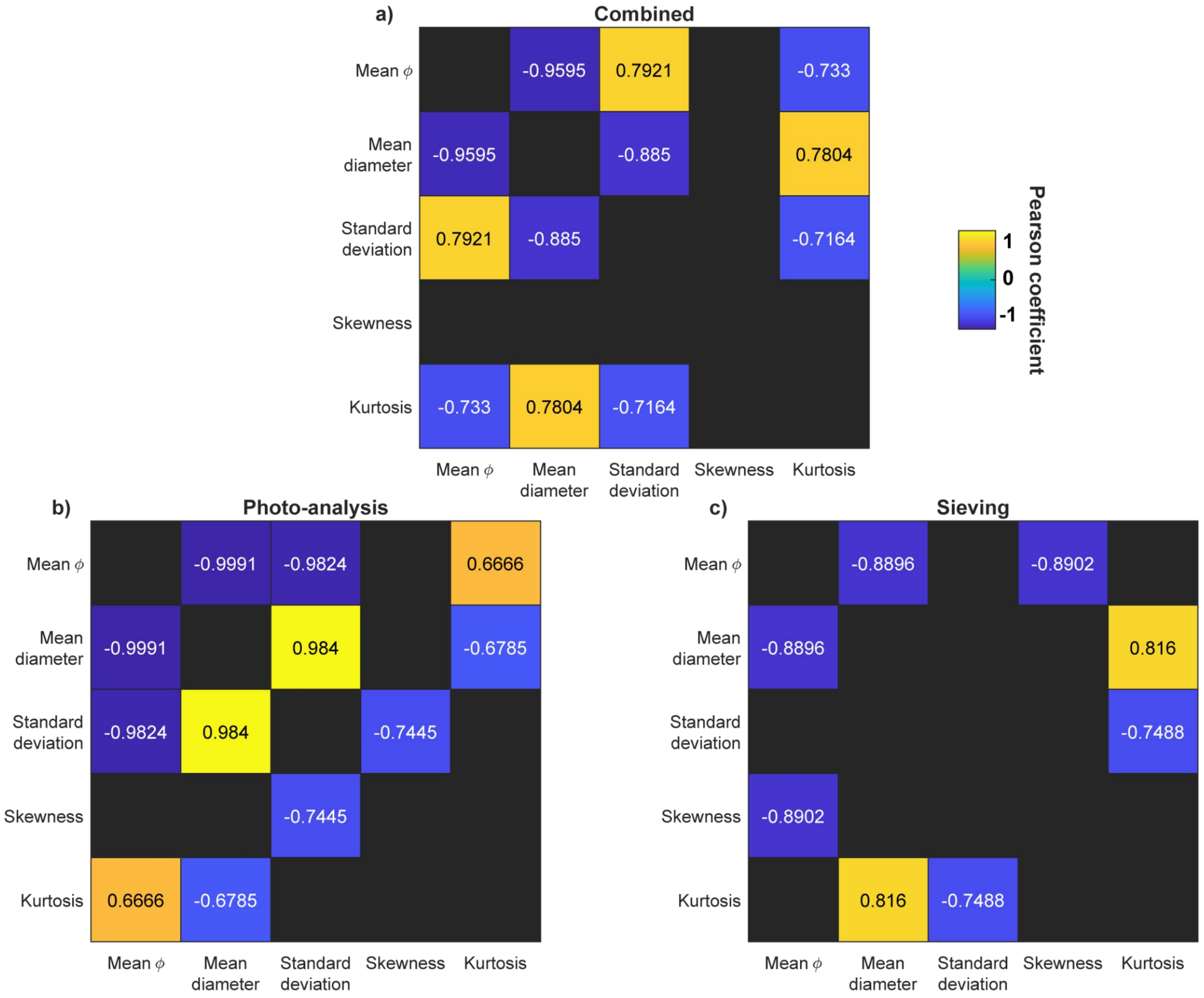

Fig. S2.
Pearson correlation coefficients among grain-size parameters for (A) combined, (B) photo-analysis, and (C) sieving datasets. Only statistically significant correlations ($p < 0.05$) are shown.

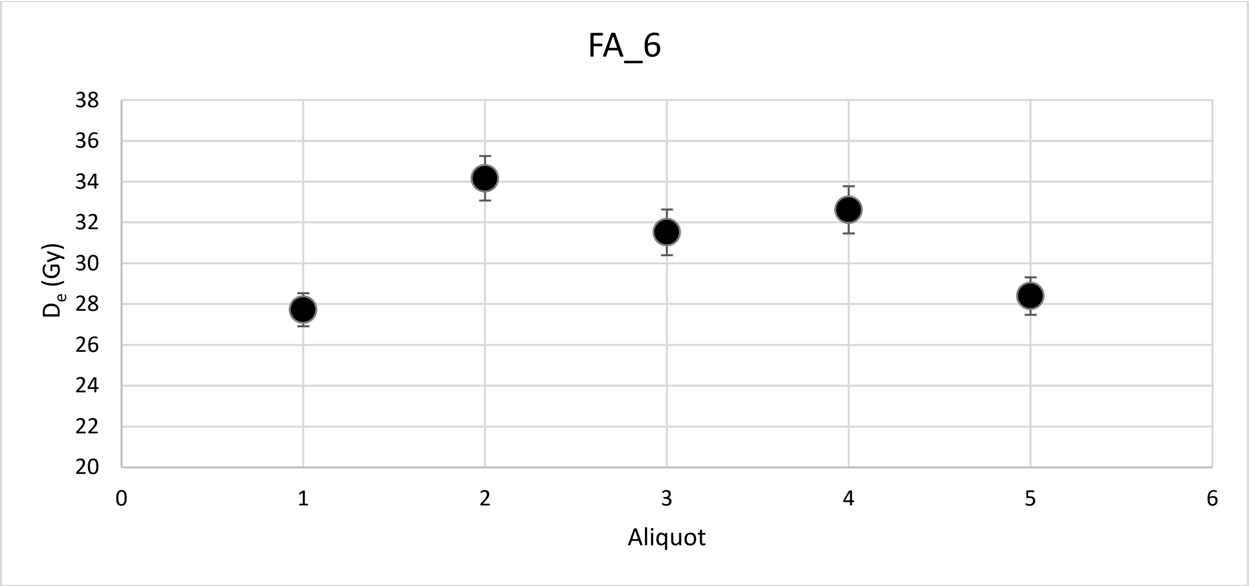

Fig. S3.
Residual dose test results on the multi-grain FA6 sample obtained after 267 min bleaching (1⌀ aliquot)

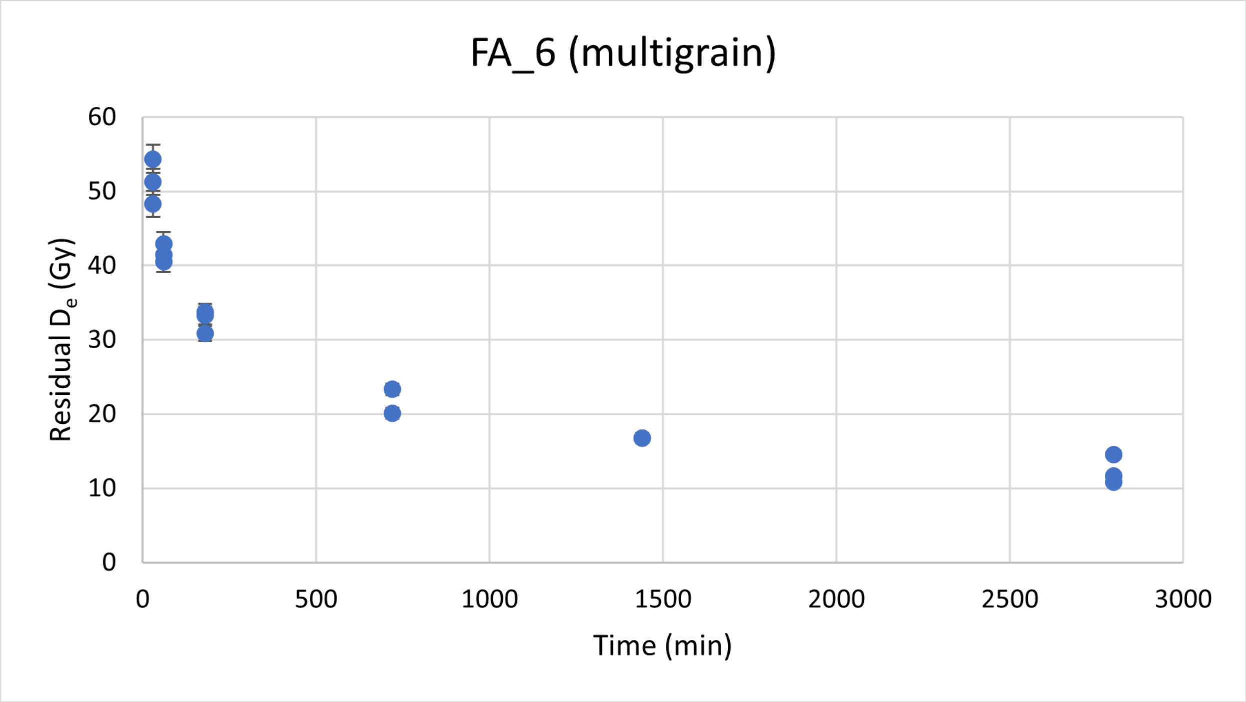

Fig. S4.
Residual dose test results on the multi-grain FA6 sample obtained for different bleaching duration.

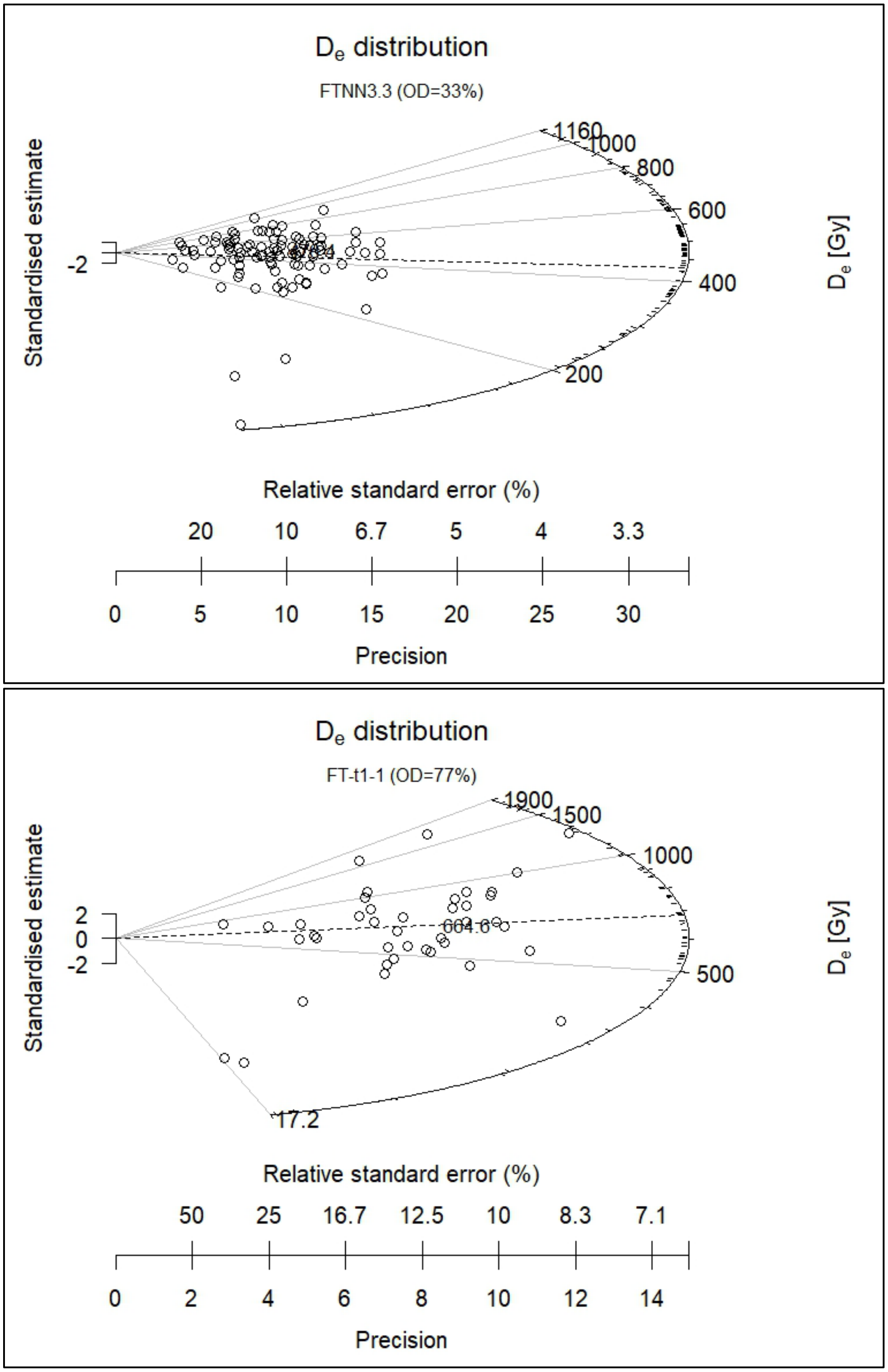

Fig. S5.
Radial plot of samples FTNN3.3 (top) and FTT11 (bottom).

Terrace	Numeric ages from Quaternary fluvial deposits of the Marche Apennines				
name	basin	source	Age (ka)	MIS	Methodology
-	Fr	(36)*	750±260	19	$^{26}Al/^{10}Be$
1st level	Tr	(40)	>350	10	$^{230}Th/^{234}U$
3rd level	E	(35)	>44.46**	2/3	^{14}C
3rd level	T	(39)	>44**	2/3	^{14}C
3rd level	Mt	(31)	>44**	2/3	^{14}C
3rd level	Mt	(31)	>43**	2/3	^{14}C
3rd level	E	(34)	41±4	2/3	^{14}C
3rd level	Ce	(32)	37.3±2.2	2/3	^{14}C
3rd level	Ce	(32)	37.3±2.2	2/3	^{14}C
3rd level	Ce	(32)	35.6±1.8	2/3	^{14}C
3rd level	E	(34)	32.7±1.2	2/3	^{14}C
3rd level	E	(34)	32.5±1.2	2/3	^{14}C
3rd level	Ce	(32)	32.5±1.2	2/3	^{14}C
3rd level	E	(34)	31.8±1.1	2/3	^{14}C
3rd level	Ce	(32)	31.7±1.05	2/3	^{14}C
3rd level	M	(37)	30.98±0.24	2/3	^{14}C
3rd level	E	(34)	30.2±0.9	2/3	^{14}C
3rd level	Ch	(38)	30.15±1.2	2/3	^{14}C
3rd level	M	(37)	26.81±0.29	2	^{14}C
3rd level	Ch	(38)	26.8±0.700	2	^{14}C
3rd level	M	(37)	26.54±0.09	2	^{14}C
3rd level	M	(37)	25.26±0.21	2	^{14}C
3rd level	E	(34)	23.5±0.4	2	^{14}C
3rd level	F	(30)	23.5±0.12	2	^{14}C
3rd level	M	(37)	23.02±0.17	2	^{14}C
3rd level	E	(35)	21.9±0.3	2	^{14}C
3rd level	T	(39)	20.02±0.15	2	^{14}C
3rd level	M	(37)	16.73±0.09	2	^{14}C
3rd level	E	(33)	15.25±0.16	2	^{14}C
3rd level	E	(33)	14.7±0.15	2	^{14}C

Table S1.

Numeric ages from Quaternary fluvial deposits of the Marche Apennines. Basin abbreviations: Ce Cesano, Ch Chienti, E Esino, M Musone, Mt Metauro, T Tenna, F Foglia, Fr Frasassi Gorge, Tr Tronto. **Chronological measurement of cosmogenic nuclide ^{26}Al:^{10}Be burial age for sediments in Grotta della Madonna - Frasassi Gorge (Ancona, Italy). ** On the limit result for AMS radiocarbon dating.*

Step	*Treatment*
1	Given dose
2	Preheat (320°C for 60 s)
3	IR stimulation for 200 s at 50°C
4 (L_x)	IR stimulation for 200 s at 290°C
5	Given test dose
6	Preheat (320°C for 60 s)
7	IR stimulation for 200 s at 50°C
8 (T_x)	IR stimulation for 200 s at 290°C
9	IR stimulation for 60 s at 325°C
10	Return to 1

Table S2.
pIRIR$_{290}$ SAR measurement protocol applied to K-feldspar grains (Thiel et al., 2011) (*89*)

River	Level	Sample #	Radiocarbon age (ka)	AMS $\delta^{13}C*10^3$	Calibrated 2 sigma age (P=95.4%)	Age (ka)
Tenna	3rd	FTN T4	34.474 (0.187)	-20 (1)	38.096BC-37.283BC	39.640 ± 0.407

Table S3.
Obtained ^{14}C ages.

	U (ppm)	Th (ppm)	K (%)	*In situ* gamma dose rate (μGy/a)
FTNN 3.5	0.676 ± 0.019	5.137 ± 0.071	0.597 ± 0.016	267 ± 6
FTNN 3.4	1.435 ± 0.031	5.110 ± 0.083	1.021 ± 0.025	263 ± 6
FTNN 3.3	1.603 ± 0.031	5.741 ± 0.084	1.076 ± 0.025	583 ± 14
FTNN 3.2	1.346 ± 0.030	2.725 ± 0.062	1.372 ± 0.030	386 ± 9
FT_T1_1	1.923 ± 0.032	5.736 ± 0.081	1.114 ± 0.025	669 ± 16
FA_6	1.059 ± 0.026	3.551 ± 0.067	0.951 ± 0.024	127 ± 3
FA_3	1.009 ± 0.023	3.311 ± 0.058	0.804 ± 0.020	433 ± 10
FA_1B	1.804 ± 0.036	5.943 ± 0.093	1.151 ± 0.027	331 ± 8

Table S4.

U, Th, and K content determined using a spectrometer with broad energy Ge (BEGe) detector and in-situ gamma dose rates measured with a portable gamma-ray spectrometer connected to a LaBr probe (Inspector 1000, Cranberra).

	FTNN 3.2	FTNN 3.3	FTNN 3.4	FTNN 3.5	FA 3	FA 1B	FA 6	FT T1 1
**Number of grains measured**	500	500	500	400	500	500	300	500
**Number of grains that passed criteria**	179	161	162	163	166	178	139	109
**Number of rejected grains**	98	62	9	114	106	40	82	68
● No intersection	12	6	2	7	21	4	7	12
● Extrapolated grains	16	4	0	25	12	6	12	15
● Saturated grains	70	52	7	82	73	30	63	41
**Accepted grains**	81	99	153	49	60	138	57	41
**Grain size (µm)**	100–120	100–120	100–120	140-200	100–120	100–120	100–120	100–120

Table S5.

Statistics on the selected grains.
